# Waterfowl Move Less in Heterogeneous and Human‐Populated Landscapes, With Implications for Spread of Avian Influenza Viruses

**DOI:** 10.1111/ele.70265

**Published:** 2026-01-23

**Authors:** Claire S. Teitelbaum, Diann J. Prosser, Joshua T. Ackerman, Sakib Ahmed, A. B. M. Sarowar Alam, Kazi Zenifar Azmiri, Nyambaya Batbayar, Joël Bêty, Abigail Blake‐Bradshaw, Dmitrijs Boiko, Nelleke H. Buitendijk, Jeffrey J. Buler, David Cabot, Michael L. Casazza, Bradley Cohen, Batmunkh Davaasuren, Sébastien Farau, Jamie Feddersen, John Fieberg, Wolfgang Fiedler, Peter Glazov, Larry R. Griffin, Matthieu Guillemain, Heath Hagy, Matthew J. Hardy, Cory Highway, David Hoffman, Tehan Kang, Allison Keever, Jennifer Kilburn, Andrea Kölzsch, Helmut Kruckenberg, Toni Laaksonen, Brian S. Ladman, Hansoo Lee, Siwan Lee, Josée Lefebvre, Pierre Legagneux, Hans Linssen, Jesper Madsen, Nicholas M. Masto, Scott McWilliams, Tori Mezebish Quinn, Carl Mitchell, Axelle Moreau, Gerhard Müskens, Scott Newman, Bart A. Nolet, Rascha J. M. Nuijten, Jay Osenkowski, Cory T. Overton, Antti Piironen, Betty Plaquin, Andrew M. Ramey, Jean Rodrigue, David Rodrigues, Kees H. T. Schreven, Yali Si, Jeffery D. Sullivan, John Takekawa, Philippe J. Thomas, Mariëlle van Toor, Jonas Waldenström, Christopher K. Williams, David W. Wolfson, Fei Xu, Ian G. Brosnan, Susan E. W. De La Cruz

**Affiliations:** ^1^ NASA Ames Research Center Moffett Field California USA; ^2^ Bay Area Environmental Research Institute Moffett Field California USA; ^3^ U.S. Geological Survey, Georgia Cooperative Fish & Wildlife Research Unit, Warnell School of Forestry & Natural Resources University of Georgia Athens Georgia USA; ^4^ U.S. Geological Survey Eastern Ecological Science Center Laurel Maryland USA; ^5^ U.S. Geological Survey Western Ecological Research Center Dixon California USA; ^6^ International Union for Conservation of Nature, IUCN Bangladesh Country Office Dhaka Bangladesh; ^7^ Wildlife Science and Conservation Center of Mongolia Ulaanbaatar Mongolia; ^8^ Centre d'études nordiques, Département de biologie Université du Québec à Rimouski Rimouski Quebec Canada; ^9^ Tennessee Technological University, College of Arts and Sciences Cookeville Tennessee USA; ^10^ Latvian National Museum of Natural History Riga Latvia; ^11^ Faculty of Medicine and Life Sciences University of Latvia Riga Latvia; ^12^ Department of Animal Ecology Netherlands Institute of Ecology Wageningen the Netherlands; ^13^ Institute for Biodiversity and Ecosystem Dynamics, Department of Theoretical and Computational Ecology University of Amsterdam Amsterdam the Netherlands; ^14^ Department of Entomology and Wildlife Ecology University of Delaware Newark Delaware USA; ^15^ School of Biological, Earth and Environmental Sciences University College Cork Cork Ireland; ^16^ Fédération Départementale des Chasseur de la Vendée La Roche‐sur‐Yon France; ^17^ Tennessee Wildlife Resources Agency Nashville Tennessee USA; ^18^ University of Minnesota St. Paul Minnesota USA; ^19^ Department of Migration Max Planck Institute of Animal Behavior Radolfzell Germany; ^20^ Institute of Geography, Russian Academy of Sciences Moscow Russia; ^21^ ECO‐LG Ltd Mabie UK; ^22^ Wildfowl & Wetlands Trust Slimbridge UK; ^23^ Office Français de la Biodiversité Arles France; ^24^ Habitat and Population Evaluation Team U.S. Fish and Wildlife Service Bismarck North Dakota USA; ^25^ Iowa Department of Natural Resources Clear Lake Iowa USA; ^26^ KoEco Daejeon Republic of Korea; ^27^ Division of Fish and Wildlife Rhode Island Department of Environmental Management Providence Rhode Island USA; ^28^ Department of Ecology, Radboud Institute for Biological and Environmental Sciences Radboud University Nijmegen the Netherlands; ^29^ Institute for Waterbird and Wetlands Research (IWWR) e.V. Verden Germany; ^30^ Department of Biology University of Turku Turku Finland; ^31^ Department of Animal and Food Sciences University of Delaware Newark Delaware USA; ^32^ Canadian Wildlife Service, Environment and Climate Change Canada Québec QC Canada; ^33^ Centre de la Science de la Biodiversité du Québec, Centre d'études nordiques, Département de biologie Université Laval Québec QC Canada; ^34^ Department of Ecoscience Aarhus University Aarhus Denmark; ^35^ University of Rhode Island Kingston Rhode Island USA; ^36^ Wageningen Environmental Research Wageningen University & Research Wageningen the Netherlands; ^37^ Food and Agriculture Organization of the United Nations Rome Italy; ^38^ Future For Nature Foundation Arnhem the Netherlands; ^39^ Wildlife Ecology and Conservation Group Wageningen University Wageningen the Netherlands; ^40^ University of Turku Turku Finland; ^41^ U.S. Geological Survey Alaska Science Center Anchorage Alaska USA; ^42^ Canadian Wildlife Service Environment and Climate Change Canada Québec QC Canada; ^43^ Coimbra College of Agriculture Polytechnic University of Coimbra Coimbra Portugal; ^44^ Institute of Environmental Sciences Leiden University Leiden the Netherlands; ^45^ Suisun Resiource Conservation District Suisun City California USA; ^46^ Environment and Climate Change Canada, National Wildlife Research Centre Carleton University Ottawa Ontario Canada; ^47^ Linnaeus University Kalmar Sweden; ^48^ Key Laboratory of the Three Gorges Reservoir Region's Eco‐Environment, Ministry of Education Chongqing University Chongqing China; ^49^ U.S. Geological Survey Western Ecological Research Center Moffett Field California USA

**Keywords:** animal movement, avian influenza, dispersal, land cover, vegetation, waterbirds, weather

## Abstract

Animal movements contribute to the spread of infectious diseases and are driven in part by environmental conditions. We investigated the links among the environment, animal movement, and infectious disease dynamics in waterfowl, which are among the primary wildlife hosts of avian influenza viruses. By combining telemetry data on 4606 individuals from 26 waterfowl species with data on land cover, weather, and vegetation, we found that waterfowl moved less in areas of higher land cover heterogeneity and higher human population density. Moreover, predicted waterfowl movement distances were weakly but positively correlated with distances between detections of H5N1 highly pathogenic avian influenza in wild waterfowl, suggesting that environmental conditions might contribute to the spread of this disease via their effects on bird movements. By considering wildlife movements alongside other drivers of infectious disease dynamics, such as livestock production and human mobility, we move closer to predicting outbreaks and informing interventions.

## Graphical abstract

We show that non‐migratory waterfowl movement distances are shorter in areas of high land cover heterogeneity and human population density, and that these movement distances are related to the spread of highly pathogenic avian influenza. These results suggest that habitat management could contribute to the spread of these viruses.

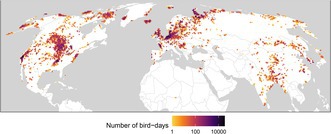

## 1 Introduction

Animal movement patterns play important roles in the spatial spread of infectious diseases, including those that threaten the health of wildlife populations. For instance, the initial spread of white‐nose syndrome, a fungal disease that has been responsible for large‐scale declines in North American bat populations, was consistent with short‐distance dispersal events of bats (Maher et al. 2012). However, relationships between movement and infection are complex; although movement contributes to the spatial spread of pathogens, it can also reduce pathogen transmission by allowing animals to escape from contaminated environments (Altizer et al. 2011). Understanding animal movement patterns and their relationships to disease is key to our ability to understand and manage diverse infectious diseases.

Many animal movements, from long‐distance seasonal migration to local foraging patterns, are closely linked to environmental conditions. For example, terrestrial mammals migrate farther in less productive landscapes (Teitelbaum et al. 2015), non‐migratory bird movements are longer in homogeneous environments (Tucker et al. 2019), butterflies fly farthest at intermediate air temperatures (Evans et al. 2019), and desert ungulate movement patterns depend on the spatial distribution of water (Nandintsetseg et al. 2019). Seasonal and spatial variation in environmental conditions and landscape structure can therefore contribute to disease dynamics by driving when and where animals move most. For example, spatial spread of rabies is slowed by the presence of rivers on the landscape (Smith et al. 2002), while increased congregation in high‐rainfall years drives anthrax outbreaks in zebra populations (Huang et al. 2021). These relationships are especially important in the context of anthropogenic climate change and land‐use change, which are altering environments, animal movements, and potentially disease dynamics globally (Gottdenker et al. 2014; Prosser et al. 2023).

Alongside livestock production, movements of waterfowl are a key driver of the dynamics of avian influenza viruses (AIVs). Wild waterfowl (ducks, geese, and swans: order Anseriformes) are among the natural hosts of low pathogenic (LP) AIVs. Non‐migratory waterfowl populations can maintain LPAIV transmission year‐round (Brown et al. 2013), while long‐distance seasonal migrations contribute to viral dispersal and reassortment (Endo and Nishiura 2018; Hill et al. 2016). Although highly pathogenic (HP) AIVs first evolved in poultry (Dhingra et al. 2018), waterfowl abundance and migration have since been linked to HPAIV outbreaks in poultry (McDuie et al. 2024; Velkers et al. 2021). These links are especially important given that recent outbreaks of the currently‐circulating H5N1 HPAIV (clade 2.3.4.4b) have been longer‐lasting and have caused increased mortality in wild birds and severe economic impacts in the poultry sector (Harvey et al. 2023). Further, waterfowl are wetland‐ and cropland‐associated species; wetland degradation has left many species increasingly dependent on croplands, remnant wetlands in protected areas, and other anthropogenic habitats (Donnelly et al. 2022; Xu et al. 2021), which brings them closer to humans and livestock (McDuie et al. 2022; Muzaffar et al. 2010). Studies of individual populations show that waterfowl move more when habitat or food are limited (Highway et al. 2024; Kleyheeg et al. 2017; Legagneux et al. 2009; Xu et al. 2021), when mortality risk from hunting is low (Highway et al. 2024; McDuie et al. 2021), and in response to extreme weather events (Masto et al. 2022; McEvoy et al. 2015). If these patterns persist across species, populations, and seasons, then spatial and temporal patterns in environmental conditions could drive global patterns of AIV transmission and dispersal.

Here, we studied movements of 26 waterfowl species in the Northern Hemisphere to understand the large‐scale environmental drivers of their non‐migratory movement distances across geographies, taxa, and seasons. We studied non‐migratory movements because continental‐scale relationships between migratory movements and AIV dispersal are better understood than those for non‐migratory movements (Gunnarsson et al. 2012; Lisovski et al. 2018; Yang et al. 2024), despite the potential importance of local and regional scales for AIV dynamics (Brown et al. 2013; Stallknecht et al. 1990). For each bird, we calculated non‐migratory movement distances at 12‐h to weekly time scales, then related these movement distances to a suite of environmental variables designed to measure landscape composition (proportional cover of croplands, protected areas, and surface water), land cover diversity, vegetation (productivity and heterogeneity), human population density, and weather (temperature, wind, and precipitation). We then used these results to estimate expected waterfowl movement distances at locations of HPAIV detections in waterfowl to understand the potential contribution of movement to viral spread during the ongoing panzootic.

## 2 Methods

### 2.1 Data Compilation and Processing

Data were downloaded from Movebank using the *move2* package in R (Kranstauber et al. 2023; R Core Team 2023) for any dates prior to January 2024 and cleaned to remove spurious locations (Supporting Information: Methods). Data were sub‐sampled to an hourly fix rate if they included more frequent locations. In addition to data collected by the authors, we used open‐access data available on Movebank (USGS Alaska Science Center 2024). Tags used GPS and/or Argos systems to estimate locations. We filtered data to winter and breeding seasons only (i.e., excluding migratory movements) for subsequent analysis (Supporting Information: Methods).

### 2.2 Calculation of Movement Metrics

We calculated movement distances within three fixed time windows: 12 h, which measured commuting distances between roosting and foraging sites; 24 h, which measured local dispersal or use of new roosting or foraging sites; and 7 days, which measured local or regional dispersal to new areas. The 7‐day window also matches a common estimate for AIV shedding duration in waterfowl (Hénaux and Samuel 2011).

For all combinations of individuals and time windows (hereafter ‘bird‐windows’), we calculated maximum pairwise displacement, defined as the maximum distance between any pair of observed locations within the time window. This metric represents the farthest potential dispersal of a virion by an infected bird. We also calculated net displacement (i.e., distance between the first and last observed location) for comparison with prior work on animal movements (Tucker et al. 2019). Because fix rates (i.e., time between locational fixes) can affect movement metrics (McDuie, Casazza, Keiter, et al. 2019), we filtered data by fix rate. Based on exploratory analyses, for maximum pairwise displacement we retained bird‐windows with at least 3, 4 or 28 fixes for 12 h, 24 h, and 7 day windows, respectively. For net displacement, we included windows with fixes covering ≥ 80% of the window (Figure S1).

### 2.3 Environmental Annotation

We annotated each location with data on environmental conditions (Table 1, Figures S2 and S3). We used data as close to contemporaneous as possible; layers ranged from hourly to static. Data were extracted within a 2500 m square buffer of each point. This distance corresponds to an achievable (but uncommon) hourly movement distance and therefore the potential environmental conditions experienced by a bird between hourly fixes.

**TABLE 1:** Descriptions and data sources for environmental variables hypothesized to affect waterfowl movement distances.

Category	Variable	Ecological relevance	Data source	Spatial resolution	Temporal resolution
Vegetation	Mean enhanced vegetation index (EVI)^a^	Vegetation indices represent forage availability for herbivores (Pettorelli et al. 2011).	MODIS MOD13Q1 (Didan 2021)	250 m	16 days
	Standard deviation of EVI	Spatial vegetation heterogeneity affects bird movements (Tucker et al. 2019)	MODIS MOD13Q1 (Didan 2021)	250 m	16 days
Land cover	Land cover diversity (Shannon Diversity Index of land cover classes (Shannon and Weaver 1948))	Landscape structure can influence movement behaviour by altering distances between resources on a landscape (Fahrig 2007)	MODIS MCD12Q1 (Friedl and Sulla‐Menashe 2022); Food and Agriculture Organization (FAO)‐Land Cover Classification System (LCCS2) classification scheme	500 m	Annual
	Herbaceous croplands (proportion cover)	Herbaceous croplands are primary food sources for many waterfowl species (Fox et al. 2017)	MODIS MCD12Q1 (Friedl and Sulla‐Menashe 2022); FAO‐LCCS2 classification scheme	500 m	Annual
	Surface water (proportion cover)	Waterfowl are aquatic species, so surface water represents habitat availability	Joint Research Centre's (JRC) Global Surface Water Mapping Layers (Pekel et al. 2016)	30 m	Static^c^
Disturbance	Proportion of area protected^b^	Animals often experience less disturbance in protected areas, especially for hunted species such as waterfowl (Highway et al. 2024)	World Database on Protected Areas (WDPA) (UNEP‐WCMC and IUCN 2023)	N/A; polygons	Static
	Human population density	Disturbance from human activity can alter movement behaviour (Varner et al. 2014)	LandScan (Sims et al. 2023)	100 m	Annual
Weather	Wind speed at 10 m	Wind provides flight support or headwind (O'Neal et al. 2018)	ERA5‐Land (Copernicus Climate Change Service 2019)^d^	9 km	Hourly
	Air temperature at 2 m	Temperatures affect thermoregulation and food availability (Masto et al. 2022)	ERA5‐Land (Copernicus Climate Change Service 2019)^d^	9 km	Hourly
	Precipitation	Precipitation can impede flight (O'Neal et al. 2018)	ERA5‐Land (Copernicus Climate Change Service 2019)^d^	9 km	hourly

^a^ When calculating mean EVI, we truncated EVI at 0 (i.e., assigned values < 0 to be 0) because values < 0 are most often water (Tuanmu and Jetz 2015) and we aimed to measure terrestrial vegetation.

^b^ All protected areas were included in this calculation because metadata on protected area type was unavailable for some protected areas; this decision likely excludes some areas that are *de facto* protected (e.g., private reserves) and includes a small proportion of disturbed public lands (Milam et al. 2016).

^c^ We used the ‘maximum extent’ of surface water, that is, whether surface water was ever present in a pixel over the 1984–2021 period; ‘maximum extent’ was the only layer that was available across the entire study area (e.g., monthly surface water estimates were missing for > 25% of locations).

^d^ Areas missing from ERA5‐Land (e.g., during movements over open ocean) were supplemented with ERA5 daily data (Copernicus Climate Change Service 2024), which provides many of the same variables ~25 km resolution.

Most data were extracted using Google Earth Engine via the *rgee* package in R (Aybar 2023; Gorelick et al. 2017). Static data (WDPA) were extracted using the *terra* package in R (Hijmans 2022). Where layers are produced with some latency, we extracted the most recent available data (e.g., 2022 land cover and human population data were used for 2023 locations). For each bird‐window, we calculated the mean value of each environmental variable across all fixes within the window.

### 2.4 Statistical Analysis

We analysed relationships between movement distances and environmental covariates using generalised additive mixed‐effects models (GAMMs), implemented using the *mgcv* package in R (Wood 2006, 2011). GAMMs are conceptually similar to generalised linear mixed‐effects models, but can fit nonlinear relationships between predictor and response variables. We expected that many of the relationships we studied would be nonlinear (e.g., nutritional value peaks at intermediate vegetation greenness (Hogrefe et al. 2017)). We fit a separate model for each combination of species, window size (12 h, 24 h, and 7 days), season (winter or breeding) and movement metric (maximum pairwise displacement or net displacement). Each full model included smoothing splines for protected area cover, Shannon index of land cover diversity, crop cover, EVI, standard deviation of EVI, surface water cover, human population density, precipitation, wind speed, and air temperature. Smoothing splines for environmental variables were fit with a maximum of three knots (i.e., constrained to a quadratic or linear function). We also included parametric fixed effects for sensor type (to account for differences in location error between GPS and Argos data) and average fix rate (to account for bias in observed movement distances), and a cyclic cubic regression spline for day of year (such that December 31 and January 1 were consecutive days). We also modelled spatial effects on movement distances using a Gaussian process with a first‐order exponential correlation function. We included random intercepts for population and individual to account for repeated measures. Models were simplified to remove concurve variables and to reduce overfitting (Wood and Augustin 2002). To evaluate the models' ability to make accurate predictions outside the range of the data, we stratified each data set by population, sorted it by time, and fit models using only the first 85% of the data. The last 15% of the data were used for model validation, for which we compared relative root mean squared error (rRMSE) on the training and validation data. For full details of model fitting, refer to Supporting Information: Methods.

From each GAMM, we extracted and plotted relationships between each covariate and the dependent variable of interest. We evaluated each species‐level model at a series of 100 points across the range of each environmental variable, then calculated the mean and standard error of these predictions across species to obtain an average fitted relationship of movement distance with each environmental variable (Supporting Information: Methods). We also used environmental conditions on January 1 and July 1, 2022 to make spatial predictions of expected movement distances.

To analyse means and variation in movement‐environment relationships, we calculated two measurements of effect size for each variable: (1) net change in expected movement between the minimum and maximum values of each explanatory (i.e., environmental) variable, and (2) gross change in expected movement over the range of each explanatory variable. Predictions were made only within the range of environmental conditions observed for the focal species. The net effect provides a signed metric of a variable's effect but does not account for nonlinearity, while the gross effect provides an unsigned total effect size. For each environmental variable, we used a linear mixed‐effects model to model this effect size as a function of the species group (goose, swan, dabbling duck, diving duck, shelduck), window size, season, and movement metric. Models also included a random intercept for dataset (i.e., species‐season combination) to account for use of the same data for models across response variables and window sizes. We excluded any variables that were removed prior to model fitting (i.e., due to concurvity); these would have had an effect size of zero, but only because they were correlated with other variables. We tested for phylogenetic signal in the residuals of these models by calculating Pagel's *λ* using a phylogeny from birdtree.org (Jetz et al. 2012) and the R packages *ape* and *phytools* (Paradis and Schliep 2019; Revell 2010, 2012). Finally, we calculated marginal mean effect sizes and pairwise differences between seasonal effect sizes in each model using the *emmeans* package (Lenth 2024).

### 2.5 Analysis of HPAIV Detections

To investigate relationships between expected movement distances and HPAIV detections in waterfowl, we gathered data on locations of HPAIV detections from the United Nations Food and Agriculture Organization's Emergency Prevention System (EMPRES) database (Pinto et al. 2010) and the U.S. Department of Agriculture's (USDA) Animal and Plant Health Inspection Service (USDA Animal and Plant Health Inspection Service 2024). We filtered the datasets to include data for wild waterfowl from January 2021 (the beginning of the USDA data) to June 2024. Because the location precision of the EMPRES data varies (Farnsworth et al. 2010) and the USDA data is identified at the second‐level administrative unit (ADM‐2) level (i.e., U.S. county), we used ADM‐2 unit centroids as the geographic location for each detection.

To measure HPAIV spread, we calculated the ‘spread distance’ to reach each new location, defined as the distance of a detection from the nearest detection within the last 6 months. This 6‐month period avoids counting new detections at the same location as ‘spread’ when virus could have been present in between detections; 6 months is within the range of viral environmental persistence duration in temperate climates (Ramey et al. 2020) and assumes that HPAIV would persist through a single breeding or winter season, but not from one to the next. Because environmental persistence varies depending on environmental conditions, we also performed sensitivity analysis using 3‐month, 1‐month, and 1‐week estimates of persistence. We calculated the mean value of each environmental variable within each ADM‐2 unit with an HPAIV detection, then calculated the mean expected 7‐day movement distance in that unit (Lebarbenchon et al. 2009). We used the mean across all species to represent an ‘average’ waterfowl species, rather than limiting averages to species tracked at or near an HPAIV detection, to avoid propagating regional biases in tracking intensity to our estimates of movement distance. We also repeated this analysis using the maximum expected 7‐day movement distance to evaluate the sensitivity of our results to the movement metric. We used the seasonal model that corresponded to the month of detection (winter models for October–March, breeding models for May–August). Environmental variables were based on the mean value within 2500 m of 50 equally spaced points within an ADM‐2 unit. We also gathered data on other variables that could impact HPAIV spread: mean chicken density (from the Gridded Livestock of the World dataset (Gilbert et al. 2018)) and mean waterfowl abundance (from all waterfowl species modelled in the eBird Status & Trends dataset (Fink et al. 2022)). eBird data were accessed using the *ebirdst* package in R (Strimas‐Mackey et al. 2023). We also calculated ADM‐2 unit area because our metric of spread distance should be larger for larger administrative units.

Next, we used a generalised additive model (GAM) to investigate relationships between predicted movement distances and spread distance; we expected that, if waterfowl movements contributed to the spread of HPAIV, spread distance would be positively related to waterfowl movement distances. Because we study non‐migratory movements, we did not analyse data from HPAIV detections in primarily migratory months (April, September, and October). We included detections from the areas of highest sampling density: the contiguous United States, Europe, and Japan. The response variable was log_10_‐transformed spread distance. Predictor variables were parametric (i.e., linear) terms for expected mean waterfowl movement distance (log_10_), ADM‐2 unit area (log_10_), season, chicken density (log_10_), and waterfowl abundance (log_10_). We also included a term for continent, to account for differences in detection, reporting, and spatial patterns among regions. Finally, we included a separate smooth term for date per continent, to account for changes in distance as the epidemic progressed. We evaluated model assumptions using quantile‐quantile plots of residuals and by calculating a global Moran's I to test for spatial autocorrelation in model residuals. We calculated partial *R* ^2^ values for each term in the model by re‐fitting the model with each variable removed, then calculating the difference in *R* ^2^ between the full and reduced model.

## 3 Results

**FIGURE 1:**
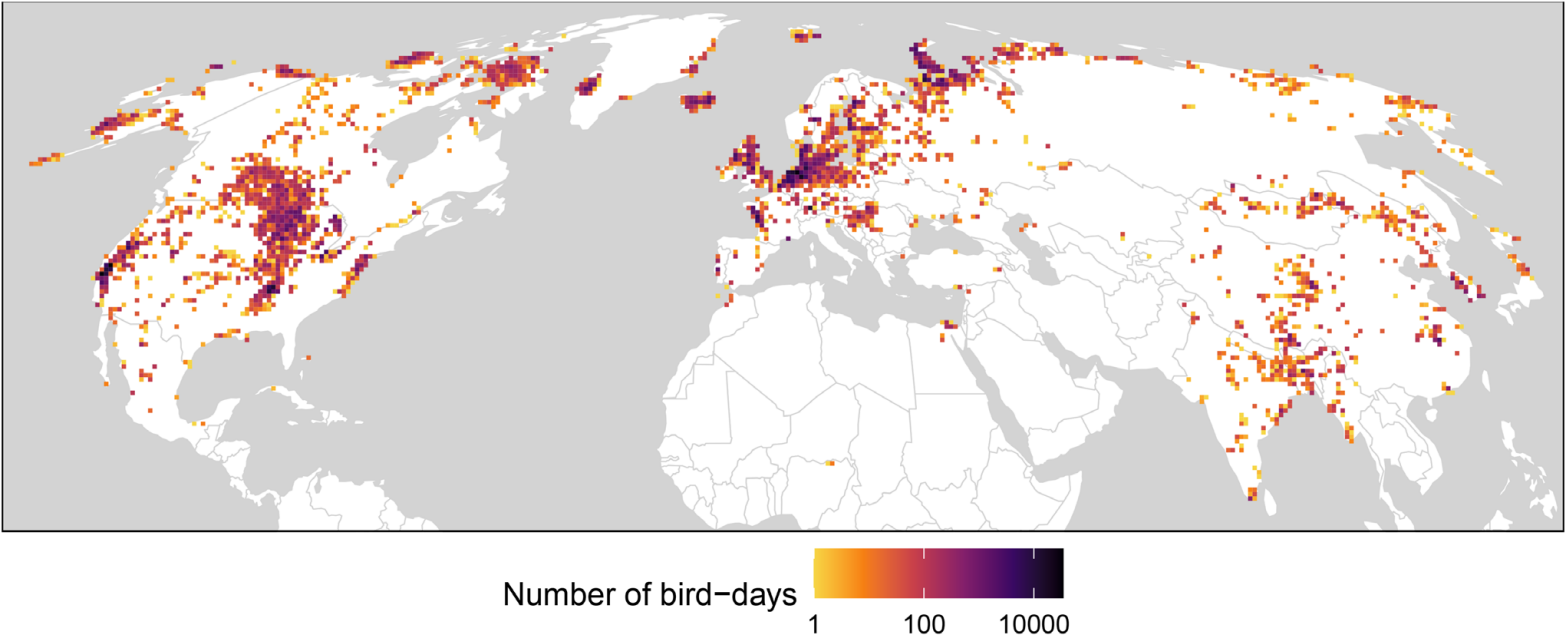
Locations and sampling intensity of tracked waterfowl during the breeding and winter seasons. Each 70‐km grid cell shows the number of bird‐days included in analyses at the 24‐h observation scale. Map in Mollweide equal‐area projection. Basemap of continental and national boundaries from Natural Earth.

We analysed GPS and Argos tracking data of 4606 individuals from 26 waterfowl species (10 dabbling duck, 3 diving duck, 1 shelduck, 9 goose and 3 swan species; Tables S1 and S2) during the breeding and winter seasons (i.e., excluding migrations) from 2003 to 2023. These data covered much of the Northern Hemisphere (18 species in the Americas, 14 in Asia, 13 in Europe, and four in Africa). Focal tracking locations were central North America, western North America, and northern Europe (Figure 1, Figure S4).

### 3.1 Data Description: Movement Metrics and Habitat

Across all species and both seasons, the median observed maximum pairwise displacement was 1.9 km within 12 h, 2.8 km within 24 h, and 7.6 km within 7 days (Table S3). Median observed net displacement distances were 1.3, 0.45, and 2.5 km, respectively. Observed movements were 2.5 times longer during winter compared to the breeding season, on average.

Species inhabited a wide range of environmental conditions; for example, within 2.5 km of observed locations, weekly mean temperatures ranged from −27°C to 43°C. Human population density ranged from 0 to 17,154 persons/km^2^. Relative to the breeding season, wintering birds generally inhabited areas with more cropland (mean crop cover of 42% vs. 15%), higher protected area cover (34% vs. 27%), higher human population density (4.7 vs. 0.06 persons/km^2^), and higher land cover diversity (mean Shannon Index of 0.68 vs. 0.54).

### 3.2 Movement‐Environment Relationships

**FIGURE 2:**
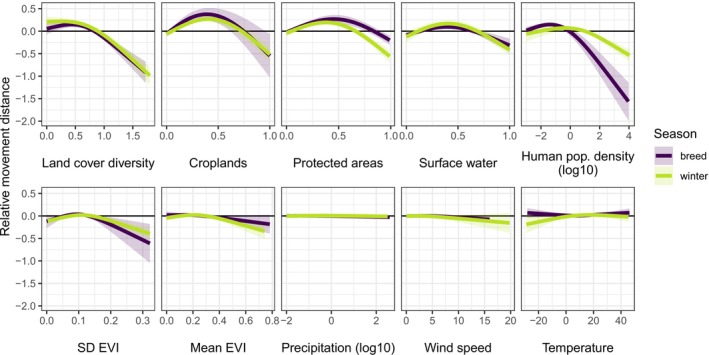
Mean fitted relationships between environmental covariates and non‐migratory movement distances across 26 waterfowl species. Results are from models of 12‐h maximum pairwise displacement. *Y*‐axes are scaled to account for species‐ and season‐specific means in movement distances, so that responses could be averaged across models. ‘Relative movement distance’ is the proportional increase or decrease in maximum pairwise displacement relative to a species' observed mean movement distance (e.g., −0.5 corresponds to a 50% reduction in movement). Shaded areas represent 95% confidence intervals of the mean across models (i.e., species). *X*‐axes span the range of conditions across all data sets for the focal season, so some model estimates are extrapolated beyond the extent of raw data for a species. EVI = Enhanced Vegetation Index.

**FIGURE 3:**
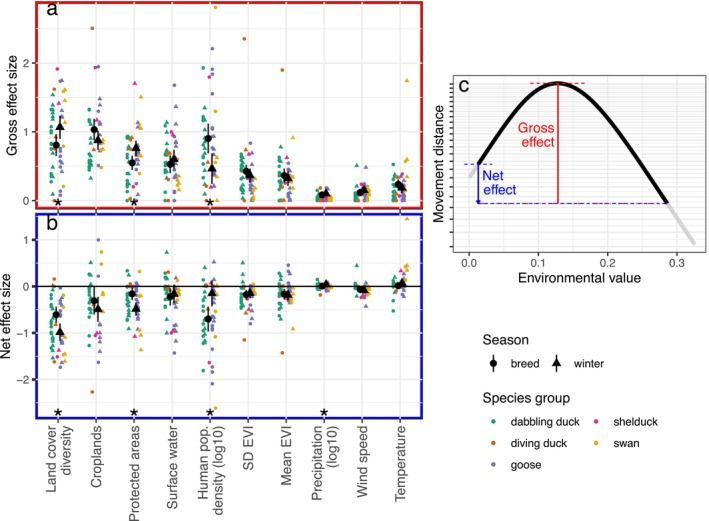
Variables measuring landscape composition and human populations have the strongest relationships with non‐migratory movement distances across 26 waterfowl species. (a) The gross effect is the maximum change in modelled movement distances across the range of an environmental variable. Large black points and error bars show the mean and 95% confidence interval of the mean for each season. Coloured points show effect sizes from individual models. Effect sizes are proportional changes (e.g., 0.5 corresponds to a 50% change in movement). Variables with significant seasonal differences in effect sizes (*p* < 0.05) are indicated with an asterisk. Results are shown for 12‐h maximum pairwise displacement; effect sizes for models using alternative time windows and movement metrics are shown in Figures S5 and S6, Tables S4 and S5. (b) Net effect size is signed and measures the proportional change in movement between the smallest and largest observed values of an environmental variable. Values < 1 represent a decrease in movement. (c) To calculate effect sizes, only values observed for a given species‐season combination (black) are included, even though other species‐season combinations may have values of the environmental variable outside this range (grey). EVI = Enhanced Vegetation Index.

In our GAMMs, land cover diversity had the largest average effect on movement distances across both seasons (breeding and winter), both movement metrics (maximum pairwise displacement and net displacement), and all three observation window sizes (12 h, 24 h, and 7 days; Figures 2 and 3, Figures S5 and S6). Gross effect sizes measure the total change in predicted movement over the range of a focal variable, while net effect sizes are signed and measure the change in predicted movement between the smallest and largest observed values of a variable (Figure 3C). Birds moved farther in areas of low land cover diversity; the mean net effect of land cover diversity across all models (i.e., seasons, metrics, and window sizes) was −0.796 (95% CI [−0.944, −0.647]), meaning that movements were 6.25 (i.e., 10^0.796^) times longer in an entirely homogeneous landscape (one land cover class within 2.5 km) than in areas with the highest land cover diversity (e.g., 8 classes in approximately equal proportions). The gross and net effects of land cover diversity were stronger in the winter than during the breeding season (net effect of −0.984 in winter vs. −0.607 in the breeding season; Figure 3, Tables S4 and S5).

Croplands, protected areas, surface water, and human population density also had significant associations with movement distances across species and seasons. Responses to croplands, protected areas, and surface water were hump‐shaped, such that the shortest movement distances occurred at very high proportion cover, but the longest movement distances occurred at low to intermediate values (35%–45% cover for all three variables). For example, average movement distances were 9.00 times farther at ~35% crop cover compared to the highest values of crop cover (mean gross effect across all models: 0.955). Birds moved less in landscapes with higher human population density (mean net effect of −0.425, i.e., 2.66 times farther in an unpopulated landscape compared to a dense urban area). Responses to human population density were stronger in the breeding season than during the winter (gross effect of 0.903 vs. 0.465), while the net effect of protected areas was stronger in the winter (net effect of −0.485 vs. −0.160).

Vegetation heterogeneity, measured using the standard deviation of EVI, had a similarly shaped, but much smaller effect than land cover diversity (mean net effect of −0.174). Vegetation productivity (mean EVI), wind speed, air temperature, and precipitation had variable and/or weak relationships with movement distances. Observed movement distances were greater for data sets with higher fix rates; for Argos (compared to GPS) devices; in mid‐winter; and at the beginning or end of the breeding season (Figures S7 and S8).

These results were consistent across movement metrics and observation window sizes (Figures S5 and S6). These models explained an average of 42% of the variation in movement distances (conditional *R* ^2^; range: 0.08–0.88, Figure S10). Models generally had moderate predictive ability on external validation data (mean rRMSE_train_ = 0.18; mean rRMSE_test_ = 0.23; Figure S9). When partitioned into fixed and random effects (i.e., environment vs. spatio‐temporal and individual‐level effects), fixed effects explained an average of 15% of the variation in movement distances (range: 0.02–0.71). Average *R* ^2^ values (both conditional and marginal) were higher for pairwise displacement than for net displacement (mean conditional *R* ^2^ of 0.49 vs. 0.34). Notably, models of 24‐h net displacement, associated with small net displacement distances and high site fidelity to roosting and/or foraging sites, had the lowest explanatory power and highest error (mean *R* ^2^ = 0.08; mean rRMSE_train_ = 0.32; mean rRMSE_test_ = 0.38; Figures S9 and S10). Models of winter movements generally performed better on validation data and had higher marginal *R* ^2^ values than did models of breeding‐season movements.

When we used our models to make predictions of movement distances across the Northern Hemisphere, we found that average expected movement distances varied about 10‐fold depending on local environmental conditions (Figure S11). The shortest predicted movement distances occurred in areas with high human population density and crop cover (India, parts of East Asia, eastern North America, and western Europe), or with high landcover diversity and large protected areas (western North America). The longest predicted movement distances occurred in areas with low landcover diversity and few protected areas (central North America and central Asia) (Figures S2 and S3).

### 3.3 HPAIV Detections in Wild Waterfowl

**FIGURE 4:**
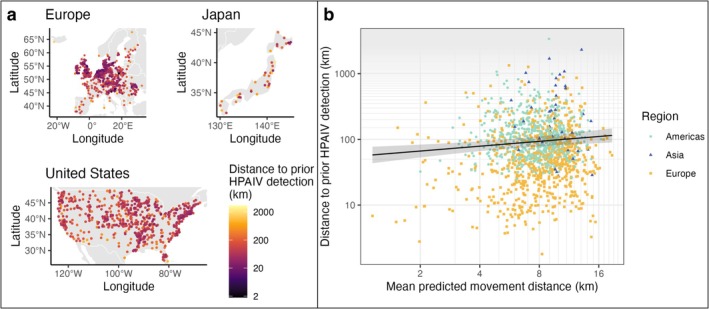
Locations of highly pathogenic avian influenza (HPAIV) detections in waterfowl, 2021–2024, are related to predicted 7‐day maximum pairwise displacement. (a) Locations of HPAIV detections in Europe, Japan and the United States. Points are the centroids of second‐level administrative units with HPAIV detections in waterfowl. Colours show ‘spread distance’, for example, the distance to the closest detection ≤ 6 months prior. Note that the three maps are at different spatial scales. (b) Relationship between spread distance and mean waterfowl movement distances, predicted from environmental conditions at detections. Points show data from individual detections, coloured by continent; line and shaded area show the modelled relationship and 95% confidence interval of the mean for the Americas from a generalised additive model, which also accounted for temporal trends, bird abundance, chicken density and administrative unit area (Table S6). Basemap of national and continental boundaries from Natural Earth.

When we used our models to predict waterfowl movement distances at known HPAIV detections based on local environmental conditions, we found that HPAIV detections in waterfowl were farther apart where predicted movement distances were longer (Figure 4, Table S6). Our model indicated that the distance between detections would be 1.76 times farther (95% CI [1.24, 2.49]) for every 10‐fold increase in weekly movement distances. The model also showed that distances between detections were greater earlier in the epidemic. Detections were closest together in Europe and farthest apart in Japan. Poultry locations could influence infection spread if waterfowl become infected via contact with farms and waterfowl abundance could amplify local transmission dynamics; accordingly, we found that detections were closer together where waterfowl abundance and chicken densities were higher. However, these effect sizes were very small (e.g., detections 0.93 times as far apart for every 10‐fold increase in waterfowl abundance). The full model explained 46.0% of the variance in distance between detections; most of this explanatory power was from variables for administrative unit area and date (partial *R* ^2^ of 12% and 8%, respectively), whereas movement distance, waterfowl abundance, and chicken density had little explanatory power (partial *R* ^2^ of < 1%). These results were qualitatively insensitive to the assumed duration of HPAIV persistence (1 week to 6 months) or the movement metric (maximum or mean movement distance; Figure S12).

## 4 Discussion

Understanding the environmental drivers of animal movements can contribute to conservation and management of wildlife, including the management of wildlife diseases. Our analysis of 26 waterfowl species indicated that human‐ and land cover‐related landscape characteristics, rather than vegetation productivity and heterogeneity or weather, had the strongest associations with waterfowl movement distances during both winter and breeding seasons. Specifically, higher land cover diversity, protected area cover and human population density within 2.5 km were associated with shorter‐distance movements across almost all species and seasons, and at 12‐h to weekly time scales. Further, the spread distance of HPAIV in wild waterfowl was weakly related to waterfowl movement distances predicted from environmental conditions. Together, these results point to a potential role of land cover in driving landscape‐scale patterns of infection by influencing animal movements.

The importance of land cover diversity and human population density implicates a combination of fragmentation, spatial structure and disturbance in driving bird movement. In heterogeneous and fragmented landscapes, the presence of smaller, more isolated habitat patches can decrease distances between habitat types and therefore reduce animals' need to move to find complementary resources (Fahrig 2007; Tucker et al. 2019). This mechanism might be particularly important for waterfowl, which fly diurnally between distinct roosting and foraging habitats (McDuie, Casazza, Overton, et al. 2019). Although higher human population densities might be expected to increase disturbance and therefore promote movement (Xu et al. 2021), movement can be constrained by human development and activity (Tucker et al. 2018). In addition, animals in areas of higher human activity are often more tolerant of disturbance (van der Kolk et al. 2024; Lowry et al. 2013). Human development can also reduce the need for local dispersal by reducing predation pressure (from natural predators and hunting) and stabilising or increasing resource availability (e.g., supplementary food, irrigation) (Eötvös et al. 2018; Teitelbaum et al. 2020; Varner et al. 2014). Many waterfowl species, including those in this study, readily tolerate less‐disturbed areas of high human population density (Champagnon et al. 2023; McKinney et al. 2006). Especially since urban landscapes are highly heterogeneous (Pickett et al. 2017), these results together suggest that landscape heterogeneity, as well as direct human presence on the landscape, are primary drivers of non‐migratory waterfowl movements at a global scale.

The net negative effects of surface water, croplands, and protected areas on movement reflect their value for waterfowl. Animals generally move less where habitat availability and/or quality is high (Bjørneraas et al. 2012); this mechanism is consistent with the stronger effects of croplands and protected areas during winter, when hunting is more prevalent and waterfowl rely more on crop‐derived foods (Fox et al. 2017; Highway et al. 2024; McDuie et al. 2021). However, for all three variables, relationships in our study were nonlinear, such that movement distances were greatest at intermediate cover. This pattern is partly a consequence of the effect of movement on habitat use: individuals that move farther experience a wider variety of landscapes and therefore intermediate cover of any given land cover type. Alternatively, intermediate cover could represent small habitat patches, which could promote movement if they provide only limited resources. Disturbance from hunting and recreation can also be highest at intermediate levels of human population density (Gutzwiller et al. 2017). These hump‐shaped patterns could also indicate functional responses to habitat availability, such that a habitat becomes more important as its availability increases (Overton and Casazza 2023). Finally, our data indicated that some birds inhabited landscapes devoid of surface water; although waterfowl, especially geese, do use upland habitats, it is unlikely that they would do so for entire days or weeks. Although the data we used are validated at the global scale (Pekel et al. 2016), this pattern suggests that errors in remote measurements of surface water could have reduced the relative importance of surface water in our models (DeVries et al. 2017). Accordingly, other variables that could serve as proxies for surface water (e.g., flooded croplands, protected wetlands) might have had increased importance in our models. More precise, direct measurements of disturbance, surface water, and crop types could therefore refine our understanding of the mechanisms by which land cover affects bird movement.

In contrast to prior macroecological studies of animal movement (Teitelbaum et al. 2015; Tucker et al. 2019), we found relatively small effects of vegetation productivity and heterogeneity on waterfowl movement distances. Although vegetation indices are useful metrics of productivity (Pettorelli et al. 2011), they do not fully capture the suite of resources needed by wildlife. For example, wintering waterfowl often move diurnally between croplands and natural or protected wetlands (Parejo et al. 2019); both of these habitats are likely to have productive vegetation, but provide different resource types. Relationships of movement with vegetation or land cover might also be scale‐dependent if vegetation productivity and land cover vary at different spatial scales, or if birds respond to vegetation at a different temporal scale than the one we measured here (e.g., lag effects) (van Moorter et al. 2013). Similarly, although extreme weather events can affect bird movement at the individual level (Overton et al. 2021), using weather variables averaged across space and time might obscure some of these local and short‐term extremes. It is therefore important to consider scale‐specific resource needs when modelling or managing animal movement.

Reducing animals' need to move is often a goal of conservation programs, since it can reduce energy expenditure (Tomlinson et al. 2014), mortality risk (e.g., from hunting (Dufour and Ankney 1995)), and human‐wildlife conflict (Buchholtz et al. 2020). Our analysis also identified a positive relationship between waterfowl movement distance and the spread distance of HPAIV, suggesting that shorter movement distances could also reduce the risk of long‐distance pathogen spread by wildlife. This pattern is especially important because waterfowl movements are usually minimally affected by AIV infection (van Dijk et al. 2015; Teitelbaum, Casazza, et al. 2023; Teitelbaum, Masto, et al. 2023). At the same time, reduced movements can amplify disease transmission locally by increasing local densities and/or environmental contamination (Altizer et al. 2011). We saw that movement distances were shortest in areas of high human population density, which is a concerning pattern for a potentially zoonotic disease such as HPAIV. Poultry production methods and biosecurity practices are the most critical methods for managing this disease (Giacinti et al. 2024; Kuiken and Cromie 2022); although we found no important relationship between chicken density and HPAIV spread distance in wild waterfowl, poultry facilities are key locations for HPAIV evolution and spillover (Giacinti et al. 2024; Prosser et al. 2024). These results are based on assumptions of spatio‐temporally homogeneous viral persistence and omnidirectional waterfowl movements. Future work that mechanistically links waterfowl movements to HPAIV detections, for example by combining genetic data on viral relatedness (Prosser et al. 2022) with simulations of waterfowl movement (e.g., based on resource selection functions (Merkle et al. 2018)), could validate the correlation we identified here and provide more precise predictions to inform wildlife and livestock management.

Beyond the HPAIV‐waterfowl system, these results show the value of linking host movement, environmental conditions and infectious disease. Although animal movements are clearly linked to disease spread (Maher et al. 2012; Smith et al. 2002), movement itself is difficult to control. Controversial measures like culling can become the only apparent options for infectious disease management, especially during an epidemic phase (e.g., chronic wasting disease in reindeer (Mysterud et al. 2020)). By quantifying how environmental conditions drive movement, it could become possible to manage movement broadly by manipulating habitat conditions, thus potentially slowing the spread of disease without the need for controversial interventions. For waterfowl, our results indicate that large protected areas that provide diverse habitat types and ample food could limit infection spread by reducing movement distances, thus adding to the established benefits of large protected areas for wildlife populations (i.e., increasing survival (Casazza et al. 2012; Guillemain et al. 2002)). These actions are especially important as rapid changes in climate and land cover are affecting animal behaviour (Tucker et al. 2018) and consequently disease dynamics (Eby et al. 2023). Linking disease to the environment via animal movement can improve our ability to protect both wildlife and humans from the risks of infectious diseases.

## Author Contributions

Claire S. Teitelbaum and Diann J. Prosser designed the study with input from Ian G. Brosnan and Susan E.W. De La Cruz. Claire S. Teitelbaum contacted data contributors and compiled data. All authors except Claire S. Teitelbaum and Ian G. Brosnan contributed to data collection through funding acquisition, study design, field work and/or supervision. Claire S. Teitelbaum performed analyses and wrote the first draft of the paper. A.B.M. Sarowar Alam, Allison Keever, Andrew M. Ramey, Antti Piironen, Bart A. Nolet, Betty Plaquin, Diann J. Prosse, David W. Wolfson, Hans Linssen, Heath Hagy, Ian G. Brosnan, Jeffery D. Sullivan, Joël Bêty, John Fieberg, Jonas Waldenström, Joshua T. Ackerman, Kees H.T. Schreven, Mariëlle van Toor, Matthew J. Hardy, Matthieu Guillemain, Michael L. Casazza, Nyambaya Batbayar, Nicholas M. Masto, Scott McWilliams, Susan E.W. De La Cruz, Toni Laaksonen and Tori Mezebish Quinn contributed to revising the approach and/or manuscript. All authors read and approved the final version of the manuscript.

## Funding

This work was supported by the Science Mission Directorate, Internet of Animals, NASA Earth Exchange.

## Supporting information


**Data S1:** ele70265‐sup‐0001‐Supinfo.zip.

## Data Availability

Data and code necessary to reproduce analyses are available at Zenodo (https://doi.org/10.5281/zenodo.14781374), including all derived data (Teitelbaum 2025). Raw data are also included, except for data with restrictions on data publication for conservation purposes. Where raw data are not included in the Zenodo repository, data are available via a reasonable request to the contact listed in Table S1. Where data are already available in public data repositories, code is provided to subset data to those used in this analysis. Potential data users are strongly encouraged to collaborate with data contacts listed in Table S1, regardless of data publication status.
