# Supplementary material for: Waterfowl Move Less in Heterogeneous and Human‐Populated Landscapes, With Implications for Spread of Avian Influenza Viruses
Source: Ecol Lett. 2026 Jan 23;29(1):e70265. doi: 10.1111/ele.70265 (PMC12829303; doi:10.1111/ele.70265)
Supplement: Supplementary file 1 — Data S1: ele70265‐sup‐0001‐Supinfo.zip. [file ELE-29-0-s001.zip › ele70265-sup-0002-Figures.docx]

**Supplementary Materials for** Waterfowl move less in heterogeneous and human-populated landscapes, with implications for spread of avian influenza viruses

Teitelbaum, Claire S.

NASA Ames Research Center, Moffett Field, CA, USA

Bay Area Environmental Research Institute, Moffett Field, CA, USA

U.S. Geological Survey, Georgia Cooperative Fish & Wildlife Research Unit, Warnell School of Forestry & Natural Resources, University of Georgia, Athens, GA, USA

ORCID: 0000-0001-5646-3184

Prosser, Diann J.

U.S. Geological Survey, Eastern Ecological Science Center, Laurel, MD, USA

ORCID: 0000-0002-5251-1799

Ackerman, Joshua T.

U.S. Geological Survey, Western Ecological Research Center, Dixon, CA, USA

ORCID: 0000-0002-3074-8322

Ahmed, Sakib

International Union for Conservation of Nature, IUCN, Bangladesh Country Office

ORCID: 0009-0004-1626-3427

Alam, A B M Sarowar

International Union for Conservation of Nature, IUCN, Bangladesh Country Office

Azmiri, Kazi Zenifar

International Union for Conservation of Nature, IUCN, Bangladesh Country Office

ORCID: 0009-0009-9482-4409

Batbayar, Nyambaya

Wildlife Science and Conservation Center of Mongolia, Ulaanbaatar, Mongolia

ORCID: 0000-0002-9138-9626

Bêty, Joël

Centre d’études nordiques, Département de biologie, Université du Québec à Rimouski, Rimouski, QC, Canada

ORCID: 0000-0002-8775-6411

Blake-Bradshaw, Abigail

Tennessee Technological University, College of Arts and Sciences, Cookeville, TN, USA

ORCID: 0000-0002-6224-3687

Boiko, Dmitrijs

Latvian National Museum of Natural History, Riga, Latvia

Faculty of Medicine and Life Sciences, University of Latvia, Riga, Latvia

Buitendijk, Nelleke H.

Netherlands Institute of Ecology, Department of Animal Ecology, Wageningen, Netherlands

University of Amsterdam, Institute for Biodiversity and Ecosystem Dynamics, Department of Theoretical and Computational Ecology, Amsterdam, Netherlands

ORCID: 0000-0002-4476-485X

Buler, Jeffrey J.

University of Delaware, Department of Entomology and Wildlife Ecology, Newark, DE, USA

ORCID: 0000-0002-2696-847X

Cabot, David

University College Cork, School of Biological, Earth and Environmental Sciences, Ireland

Casazza, Michael L.

U.S. Geological Survey, Western Ecological Research Center, Dixon, CA, USA

ORCID: 0000-0002-5636-735X

Cohen, Bradley

Tennessee Technological University, College of Arts and Sciences, Cookeville, TN, USA

Davaasuren, Batmunkh

Wildlife Science and Conservation Center of Mongolia, Ulaanbaatar, Mongolia

ORCID: 0000-0002-8496-1508

Farau, Sébastien

Fédération Départementale des Chasseur de la Vendée, La Roche-sur-Yon, France

ORCID: 0000-0003-3592-2446

Feddersen, Jamie

Tennessee Wildlife Resources Agency, Nashville, TN, USA

Fieberg, John

University of Minnesota, St. Paul, MN, USA

ORCID: 0000-0002-3180-7021

Fiedler, Wolfgang

Max Planck Institute of Animal Behavior, Department of Migration, Radolfzell, Germany

ORCID: 0000-0003-1082-4161

Glazov, Peter

Institute of Geography, Russian Academy of Sciences, Moscow, Russia

ORCID: 0000-0003-3462-7031

Griffin, Larry R.

ECO-LG Ltd, Mabie, UK

Wildfowl & Wetlands Trust, Slimbridge, UK

ORCID: 0000-0003-4472-9883

Guillemain, Matthieu

Office Français de la Biodiversité, La Tour du Valat, Arles, France

ORCID: 0000-0002-0354-771X

Hagy, Heath

U.S. Fish and Wildlife Service, Habitat and Population Evaluation Team, Bismarck, ND, USA

Hardy, Matthew J.

University of Delaware, Department of Entomology and Wildlife Ecology, Newark, DE, USA

ORCID: 0000-0001-7574-4790

Highway, Cory

Tennessee Technological University, College of Arts and Sciences, Cookeville, TN, USA

ORCID: 0009-0001-2609-3677

Hoffman, David

Iowa Department of Natural Resources, Clear Lake, IA, USA

Kang, Tehan

KoEco, Daejeon, Republic of Korea

Keever, Allison

Tennessee Technological University, College of Arts and Sciences, Cookeville, TN, USA

ORCID: 0000-0002-5194-3987

Kilburn, Jennifer

Rhode Island Department of Environmental Management, Division of Fish and Wildlife, Providence, RI, USA

Kölzsch, Andrea

Department of Migration, Max Planck Institute of Animal Behavior, Radolfzell, Germany

Department of Ecology, Radboud Institute for Biological and Environmental Sciences, Radboud Univeristy, Nijmegen, Netherlands

ORCID: 0000-0003-0193-1563

Kruckenberg, Helmut

Institute for Waterbird and Wetlands Research (IWWR) e.V., Verden, Germany

ORCID: 0000-0003-3840-1240

Laaksonen, Toni

Deaprtment of Biology, University of Turku, Turku, Finland

ORCID: 0000-0001-9035-7131

Ladman, Brian S.

University of Delaware, Department of Animal and Food Sciences, Newark, DE, USA

ORCID: 0000-0003-2696-5145

Lee, Hansoo

KoEco, Daejeon, Republic of Korea

Lee, Siwan

KoEco, Daejeon, Republic of Korea

Lefebvre, Josée

Canadian Wildlife Service, Environment and Climate Change Canada, Québec, QC, Canada

ORCID: 0000-0001-8399-5776

Legagneux, Pierre

Centre de la Science de la Biodiversité du Québec, Centre d’études nordiques, Département de biologie, Université Laval, Québec, QC, Canada

Linssen, Hans

Netherlands Institute of Ecology, Department of Animal Ecology, Wageningen, Netherlands

University of Amsterdam, Institute for Biodiversity and Ecosystem Dynamics, Department of Theoretical and Computational Ecology, Amsterdam, Netherlands

ORCID: 0000-0002-0447-5092

Madsen, Jesper

Aarhus University, Department of Ecoscience, Aarhus, Denmark

ORCID: 0000-0003-3246-0215

Masto, Nicholas

U.S. Fish and Wildlife Service, Habitat and Population Evaluation Team, Bismarck, ND, USA

McWilliams, Scott

University of Rhode Island, Kingston, RI, USA

ORCID: 0000-0002-9727-1151

Mezebish Quinn, Tori

University of Rhode Island, Kingston, RI, USA

ORCID: 0000-0001-6187-7365

Mitchell, Carl

Wildfowl & Wetlands Trust, Slimbridge, UK

Moreau, Axelle

Fédération Départementale des Chasseur de la Vendée, La Roche-sur-Yon, France

ORCID: 0000-0001-7551-6771

Müskens, Gerhard

Wageningen Environmental Research, Wageningen University & Research, Wageningen, Netherlands

Newman, Scott

Food and Agriculture Organization of the United Nations, Rome, Italy

Nolet, Bart A.

Netherlands Institute of Ecology, Department of Animal Ecology, Wageningen, Netherlands

University of Amsterdam, Institute for Biodiversity and Ecosystem Dynamics, Department of Theoretical and Computational Ecology, Amsterdam, Netherlands

ORCID: 0000-0002-7437-4879

Nuijten, Rascha J.M.

Future For Nature Foundation, Arnhem, the Netherlands

Wildlife Ecology and Conservation Group, Wageningen University, Wageningen, Netherlands

Osenkowski, Jay

Rhode Island Department of Environmental Management, Division of Fish and Wildlife, Providence, RI, USA

Overton, Cory T.

U.S. Geological Survey, Western Ecological Research Center, Dixon, CA, USA

ORCID: 0000-0002-5060-7447

Piironen, Antti

University of Turku, Turku, Finland

ORCID: 0000-0003-1986-9593

Plaquin, Betty

Office Français de la Biodiversité, La Tour du Valat, Arles, France

Ramey, Andrew M.

U.S. Geological Survey, Alaska Science Center, Anchorage, AK, USA

ORCID: 0000-0002-3601-8400

Rodrigue, Jean

Canadian Wildlife Service, Environment and Climate Change Canada, Québec, QC, Canada

ORCID: 0009-0001-7456-7427

Rodrigues, David

Polytechnic University of Coimbra, Coimbra College of Agriculture, Coimbra, Portugal

ORCID: 0000-0002-3855-7686

Schreven, Kees H.T.

Netherlands Institute of Ecology, Department of Animal Ecology, Wageningen, Netherlands

University of Amsterdam, Institute for Biodiversity and Ecosystem Dynamics, Department of Theoretical and Computational Ecology, Amsterdam, Netherlands

ORCID: 0000-0002-0550-7447

Si, Yali

Leiden University, Institute of Environmental Sciences, Leiden, Netherlands

ORCID: 0000-0001-7107-9850

Sullivan, Jeffery D.

U.S. Geological Survey, Eastern Ecological Science Center, Laurel, MD, USA

ORCID: 0000-0002-9242-2432

Takekawa, John

Suisun Resource Conservation District, Suisun City, CA, USA

Thomas, Philippe J.

Environment and Climate Change Canada, National Wildlife Research Centre, Carleton University, Ottawa, ON, Canada

van Toor, Mariëlle

Linnaeus University, Kalmar, Sweden

ORCID: 0000-0002-2254-5779

Waldenström, Jonas

Linnaeus University, Kalmar, Sweden

ORCID: 0000-0002-1152-4235

Williams, Christopher K.

University of Delaware, Department of Entomology and Wildlife Ecology, Newark, DE, USA

Wolfson, David W.

University of Minnesota, St. Paul, MN, USA

ORCID: 0000-0003-1098-9206

Xu, Fei

Key Laboratory of the Three Gorges Reservoir Region’s Eco-Environment, Ministry of Education, Chongqing University, Chongqing, China

Brosnan, Ian G.

NASA Ames Research Center, Moffett Field, CA, USA

ORCID: 0000-0003-2509-4325

De La Cruz, Susan E.W.

U.S. Geological Survey, Western Ecological Research Center, Moffett Field, CA, USA

ORCID: 0000-0001-6315-0864

**Corresponding author:** Claire S. Teitelbaum (claire.teitelbaum@gmail.com)

## Supplementary Methods

### Data processing and calculation of movement metrics

Erroneous locations were identified using a speed filter of 150 m/s, which would correspond to very fast flight (McDuie *et al.* 2019). For devices that provided error estimates, we removed locations with errors >200 m. We removed the first three days of tracking to account for potential effects of capture on movement (Teitelbaum *et al.* 2023). We initially segmented each track into behavioral seasons: winter, breeding, spring and fall migration, and spring and fall stopovers. We first used algorithms based on movement distances and directions (Teitelbaum *et al.* 2023); we considered an animal to be migrating if it moved greater than a daily threshold distance (40 km for ducks and trumpeter swans; 80 km for geese, other swans, and northern pintail) in a seasonally relevant direction (i.e., northward January 21-July 16, southward July 30-December 31). These distance and date criteria were based on exploratory data analysis. We then manually checked and edited each classification based on plots of latitude and net displacement over time. We included potential molting periods as part of the breeding season but excluded molt migrations (i.e., movements from breeding to molting sites (Salomonsen 1968)). For non-migrants (i.e., birds with no detectable seasonal pattern of displacement), we considered the breeding season to include the months of April through September, and the winter to include October through March.

When calculating movement metrics, we defined windows based on time since the first location, after removing the first three days of tracking data. For 12-hour and 24-hour windows, we split windows at nighttime (between 23:00 and 03:00) to avoid confounding effects of time of day. In other words, 12-hour windows measured movement distances for windows beginning at nighttime and ending in daytime, so that diel movements would be captured by the metrics. 24-hour windows began and ended at night, so that net displacement measured distances between subsequent nighttime locations.

### Movement models

We fit models for species-season-window combinations with at least 90 data points (bird-windows). Each full model included smoothing splines for mean protected area cover, mean Shannon index of land cover diversity, mean crop cover, mean enhanced vegetation index (EVI), mean of the standard deviation of EVI within 2,500 m of each location, surface water cover, mean population density, mean precipitation, mean wind speed, and mean temperature across all fixes within the window. We used thin plate splines and a maximum of 3 knots for each of these effects (Wood 2003); this relatively low number of knots reduced explanatory power slightly (~5% in exploratory analyses) but increased the interpretability of models and reduced the risk of overfitting. Exploratory analyses showed no major differences in the overall shapes of response curves with more knots. If there was variation in fix rates and/or sensor types for a given species, we also included a linear (parametric) term for number of fixes (log transformed) and a categorical variable for sensor type (GPS vs. Argos), to account for the greater location error from Argos tags. All models also included a smoothed effect of day of year, which accounted for temporal autocorrelation in movement distances and changes in movement within a season. We used a cyclic cubic smooth, so that day of year 1 and day of year 365 were adjacent, and an initial 15 knots. Each model also included random intercepts for individual ID and population ID, to account for non-independence in the data. Finally, all models included a Gaussian process smooth (i.e., kriging) of X and Y coordinates for each point (i.e., and individual’s median location over the window). The spatial smooth used an initial number of knots that was 2% of the number of unique data points (or 10% of the number of data points if the number of knots would otherwise be <20), and a range that was the maximum within-population geographic distance between points. We used a first-order exponential correlation function; preliminary analyses indicated that results were insensitive to the order of the exponential function

Because we later used our models to predict movement distances at new locations (described in main text), we evaluated their predictive ability using a training/validation approach. For each species-level data set, we used the first 85% of points for each population as training data and the last 15% of points for each population as validation data. Stratifying by population ensured that the broad geographic range of each species was included in the training data, but sorting by time provided a larger degree of independence between training and validation data compared to randomly sampling points (Roberts *et al.* 2017).

We began by fitting a generalized additive model (GAM) (Wood 2006, 2011) without terms for individual ID, population ID, space, or time. We then calculated concurvity (the nonlinear equivalent of collinearity), removed variables with estimated concurvity values >0.8 (Shrestha 2020), and re-fit the model with random effects (i.e., a GAMM). For a pair of concurve variables, we removed the one with the smallest effect size in the model. Models were fit using the *bam* function, which is designed for large data sets, and parameters were estimated using fast restricted maximum likelihood (fREML). These GAMMs were fit using penalized regression splines (Wood & Augustin 2002), which we found effectively simplified the models. To evaluate assumptions of normality of residuals and independence of data points, we calculated global Moran’s *I*, using the *ape* package (Moran 1950; Paradis & Schliep 2019), to test for residual spatial autocorrelation and examined quantile-quantile plots of residuals. Because of our large sample sizes for some species, we considered a model to have acceptable fit if the global Moran’s *I* value was in [-0.2,0.2], even if the statistic was significantly different from zero (*p*<0.05). If Moran’s *I* was outside this range, we doubled the number of knots and re-fit the model. We also calculated a marginal R^2^ value as the R^2^ of a model with the terms for day of year, space, and individual ID removed, but with smoothing parameters for other variables fixed at their fitted values (Burgos & Wood 2007). Fixing smoothing parameters at their fitted values constrained the ability of the fixed effects to explain the variance originally explained by the removed spatio-temporal and individual ID terms (Burgos & Wood 2007), thus avoiding issues related to partial R^2^s and collinearity (Stoffel *et al.* 2021). Although day of year was fitted as a fixed effect, we did not include it in the calculation of the marginal R^2^ because we were primarily interested in measuring the variance explained by environmental variables. Finally, we calculated relative root mean squared error (rRMSE) on both training and validation data sets to evaluate model predictive ability:

$$rRMSE= \frac{\sqrt{\frac{1}{N}\sum_{i=1}^{N} {(y_{i}-\hat{y}_{i})}^{2}}}{\frac{1}{N}\sum_{i=1}^{N} y_{i}}$$

, where $y_{i}$ is the observed movement distance and $\hat{y}_{i}$ is the predicted movement distance. We used rRMSE instead of RMSE to allow comparisons across models with different ranges of response variables (i.e., differences in movement distances across movement metrics, species, and window sizes).

To visualize effects and compare effect sizes across models, we evaluated each model at a standard set of environmental values. For each of the 10 environmental variables, we sampled equally-spaced values across the range of the variable for all species. We then evaluated the model in two ways: first, separately for each component (type = “terms” in *mgcv*), which provided the scaled effect of each variable, and second, at the response level for each variable, with all other variables held at their median. We made predictions for GPS sensors if a data set included by GPS and Argos data. We excluded the effects of individual ID and population from these predictions. To calculate average responses across species (Fig. 2), we calculated the mean and 95% confidence interval of the predicted values. Finally, to map predicted movement distances across space, we compiled data on environmental conditions on two dates (January 1 and July 1, 2022), resampled all layers to a common grid, calculated model predictions for each species, and then calculated the mean predicted value across species. We masked each seasonal map with the combined seasonal distributions of all species included in analyses, derived from seasonal range maps from BirdLife International (BirdLife International & Handbook of the Birds of the World 2023). As in our analysis of HPAIV spread (described in main text), we used combined movement distances and range maps across all species to avoid propagating regional biases in tracking intensity to our estimates of movement distance.

*Any use of trade, product, or firm names are for descriptive purposes only and do not imply endorsement by the U.S. Government.*

### References

BirdLife International & Handbook of the Birds of the World. (2023). Bird species distribution maps of the world.

Burgos, J. & Wood, S. (2007). *[R] variance explained by each term in a GAM*. Available at: https://stat.ethz.ch/pipermail/r-help/2007-October/142811.html. Last accessed 16 August 2024.

McDuie, F., Casazza, M.L., Keiter, D., Overton, C.T., Herzog, M.P., Feldheim, C.L., *et al.* (2019). Moving at the speed of flight: Dabbling duck-movement rates and the relationship with electronic tracking interval. *Wildlife Research*, 46, 533–543.

Moran, P.A.P. (1950). Notes on Continuous Stochastic Phenomena. *Biometrika*, 37, 17–23.

Paradis, E. & Schliep, K. (2019). ape 5.0: an environment for modern phylogenetics and evolutionary analyses in R. *Bioinformatics*, 35, 526–528.

Roberts, D.R., Bahn, V., Ciuti, S., Boyce, M.S., Elith, J., Guillera-Arroita, G., *et al.* (2017). Cross-validation strategies for data with temporal, spatial, hierarchical, or phylogenetic structure. *Ecography*, 40, 913–929.

Salomonsen, F. (1968). The moult migration. *Wildfowl*, 19, 5–24.

Shrestha, N. (2020). Detecting Multicollinearity in Regression Analysis. *American Journal of Applied Mathematics and Statistics*, 8, 39–42.

Stoffel, M.A., Nakagawa, S. & Schielzeth, H. (2021). partR2: partitioning R2 in generalized linear mixed models. *PeerJ*, 9, e11414.

Teitelbaum, C.S., Casazza, M.L., McDuie, F., De La Cruz, S.E.W., Overton, C.T., Hall, L.A., *et al.* (2023). Waterfowl recently infected with low pathogenic avian influenza exhibit reduced local movement and delayed migration. *Ecosphere*, 14, e4432.

Wood, S.N. (2003). Thin Plate Regression Splines. *Journal of the Royal Statistical Society Series B: Statistical Methodology*, 65, 95–114.

Wood, S.N. (2006). *Generalized additive models: an introduction with R*. Chapman and Hall/CRC.

Wood, S.N. (2011). Fast Stable Restricted Maximum Likelihood and Marginal Likelihood Estimation of Semiparametric Generalized Linear Models. *Journal of the Royal Statistical Society Series B: Statistical Methodology*, 73, 3–36.

Wood, S.N. & Augustin, N.H. (2002). GAMs with integrated model selection using penalized regression splines and applications to environmental modelling. *Ecological Modelling*, 157, 157–177.

## Supplementary Figures and Tables

**
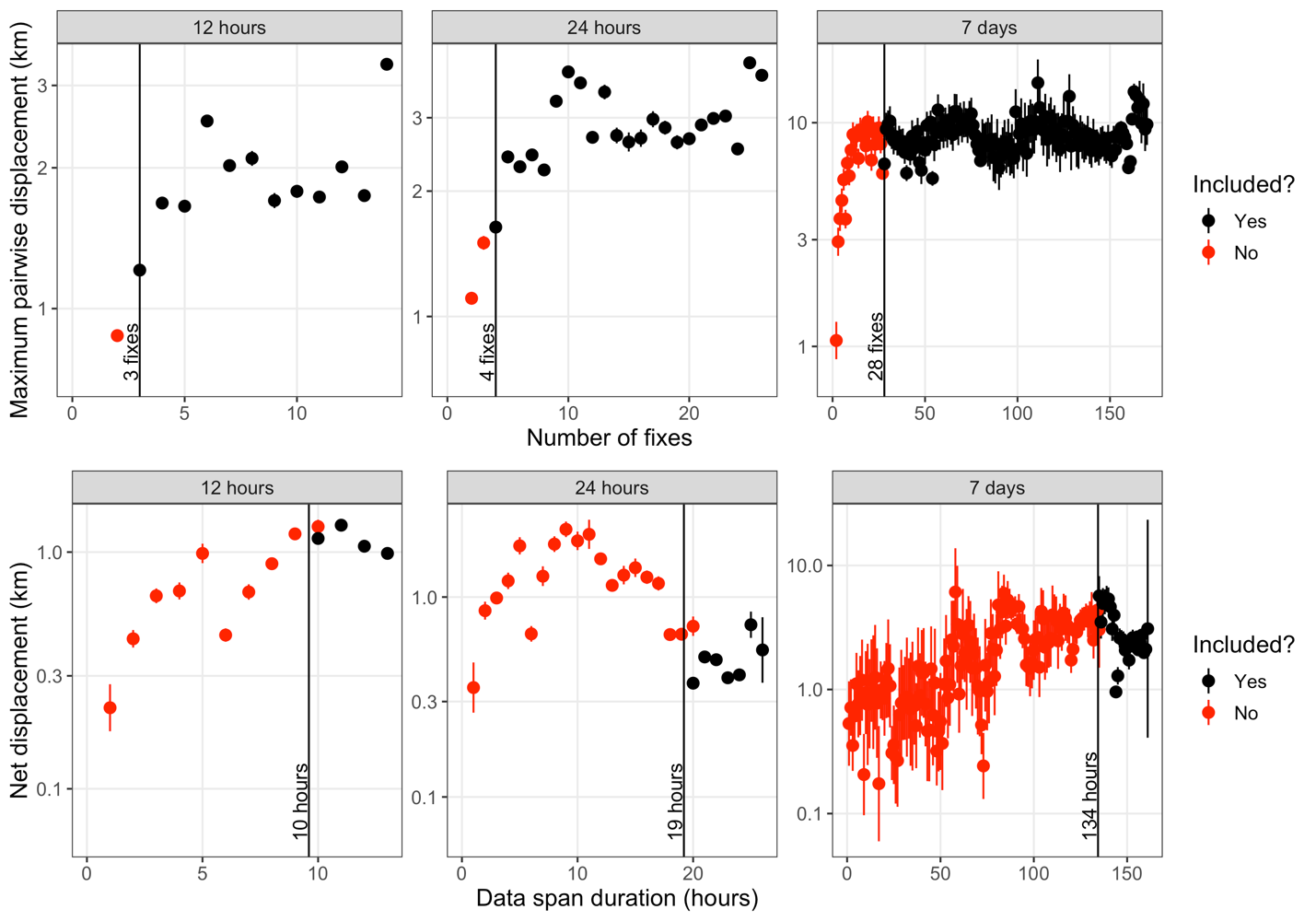
Figure S1:** Filtering criteria for movement metrics of 26 species of waterfowl during breeding and winter seasons. Points show the mean of a movement metric for all data points with a given fix rate or duration. Error bars show two standard errors of the mean. Vertical lines are the thresholds for inclusion. Red points are filtered out prior to analysis; black points are included. Pairwise displacement (top) is filtered based on average fix rates (number of locations within a window). A 4-hour fix rate (i.e., 3 fixes in 12 hours) is required for the 12-hour window and a 6-hour fix rate is required for 24-hour and 7-day windows. Net displacement (bottom) is filtered based on the time between the first and last fixes in a window; fixes must cover at least 80% of the window. Data duration is binned to integer values; red points sometimes appear to the right of the vertical line because of integer rounding. Note the log scales of the y-axes and differences in scales across panels.

***Figure S2***: Environmental covariates used to model waterfowl movement distances. Values displayed are derived from the closest data available to January 1, 2022. Total daily precipitation is log_10_-transformed and human population density is natural log-transformed. Basemap of continental boundaries from Natural Earth.

***Figure S3***: Environmental covariates used to model waterfowl movement distances. Values displayed are derived from the closest data available to July 1, 2022. Total daily precipitation is log_10_-transformed and human population density is natural log-transformed. Basemap of continental boundaries from Natural Earth.


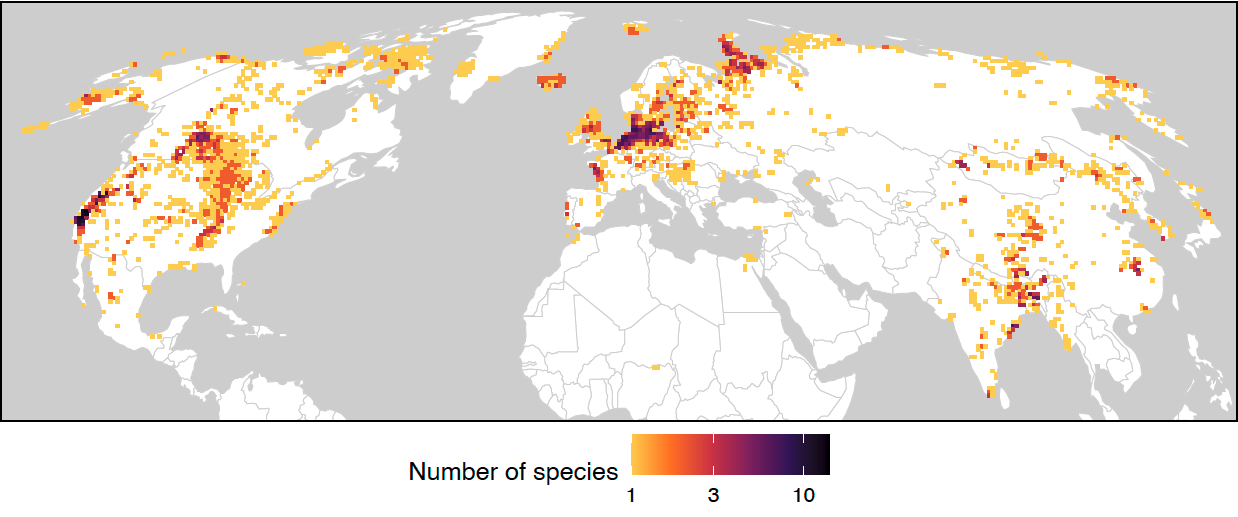


**Figure S4**: Locations of tracked waterfowl during the breeding and winter seasons. Each 70-km grid cell shows the number of species included in analyses at the 24-hour observation scale. Map in Mollweide equal-area projection. Basemap of national and continental boundaries from Natural Earth.


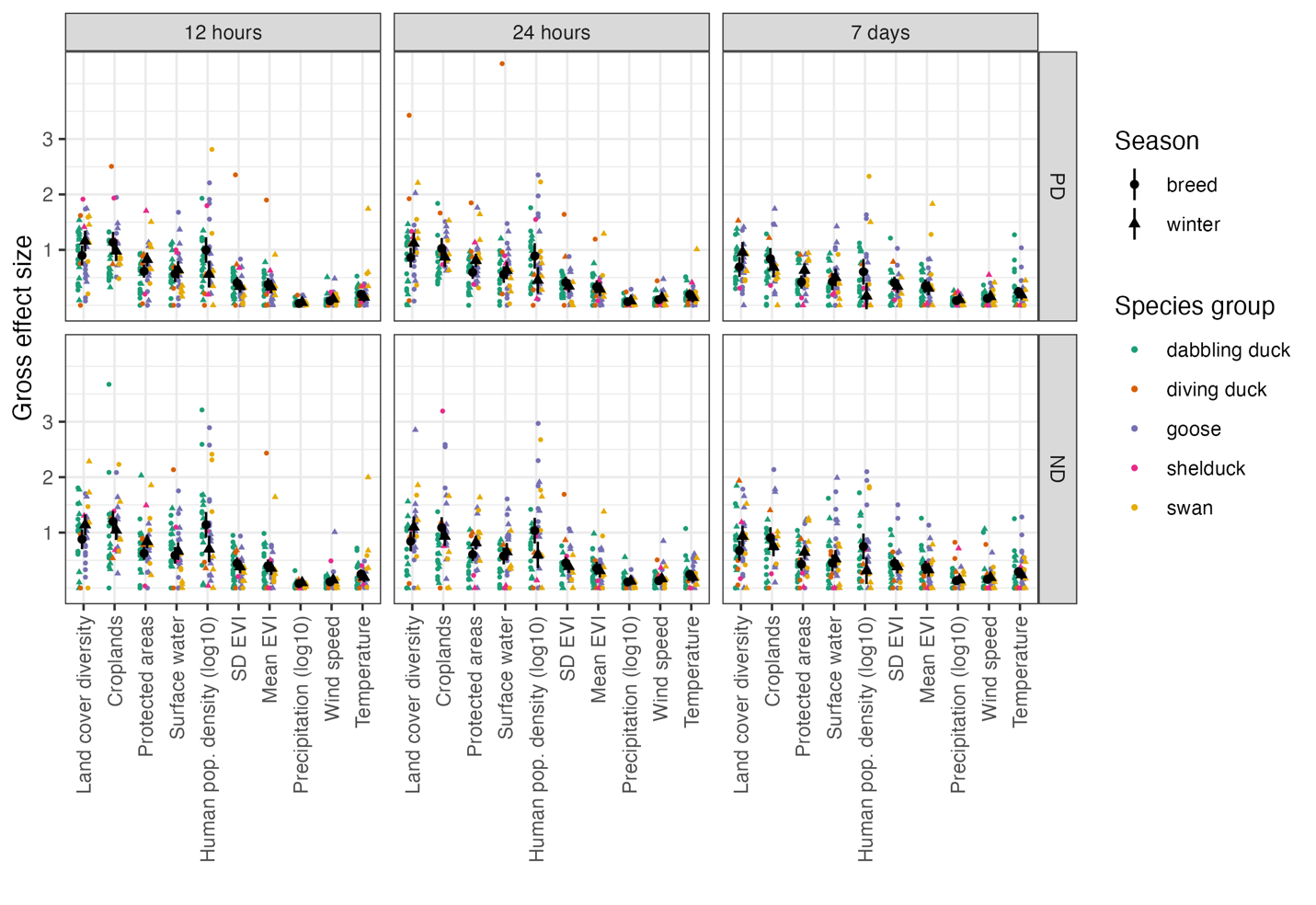


***Figure S5:*** Gross effect sizes across all models of non-migratory movement distances of 26 waterfowl species during breeding and winter seasons. Gross effect sizes measure the total change in predicted movement over the range of a focal variable (e.g., 2 represents a 200% change in displacement distance). Figure 3 in main text includes a visual representation of effect size calculation. Individual points represent results from species-level models; large black points and error bars show the mean and 95% confidence intervals across species for each season. Panels display results by window size (columns) and movement metric (rows). Abbreviations: pairwise displacement (PD); net displacement (ND); enhanced vegetation index (EVI).


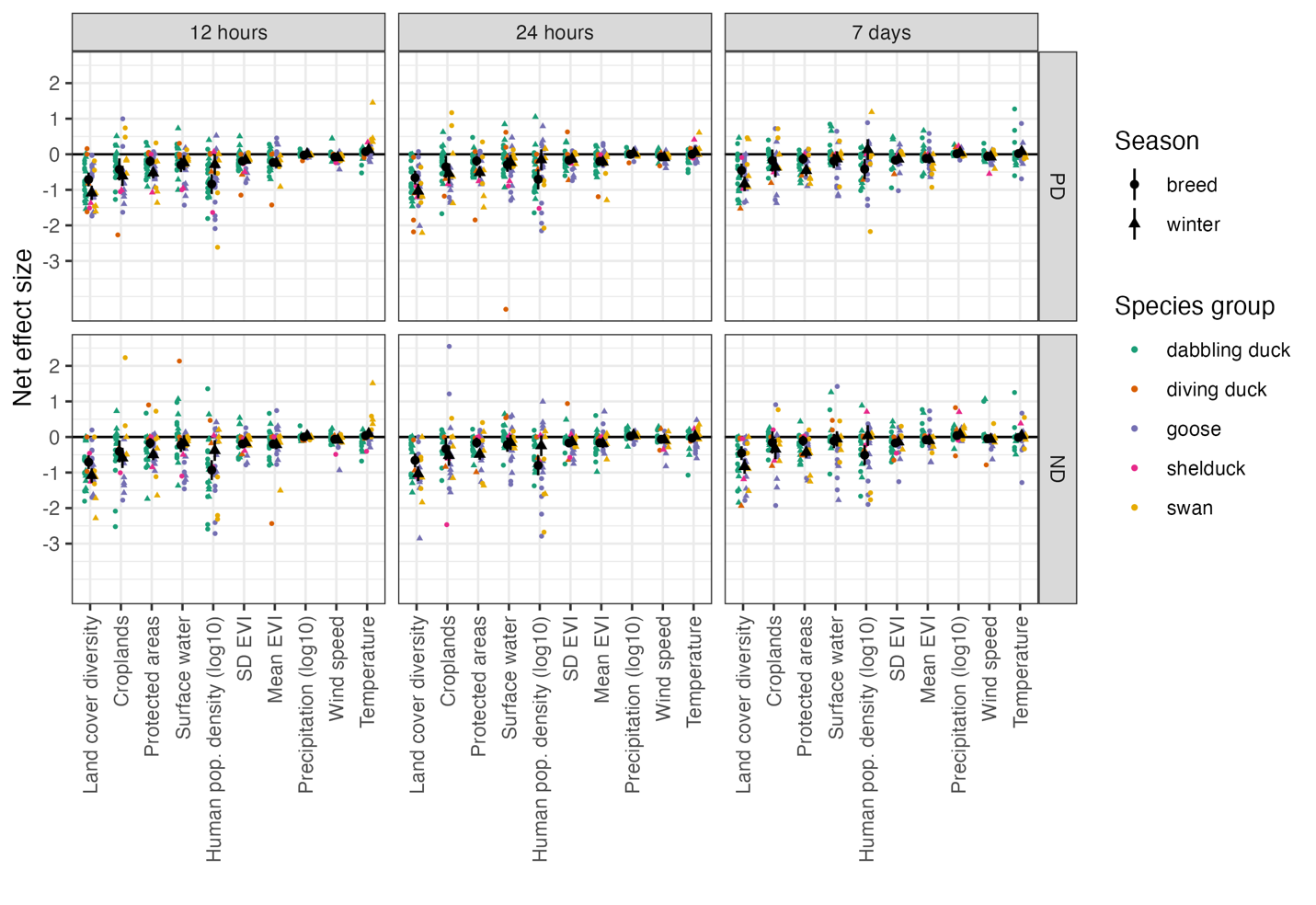


***Figure S6:*** Net effect sizes across all models of non-migratory movement distances of 26 waterfowl species during breeding and winter seasons. Net effect size is signed and measures the overall change in movement between the smallest and largest observed values of a variable (e.g., -0.5 corresponds to a 50% reduction in displacement distance). Figure 3 in main text includes a visual representation of effect size calculation. Individual points represent results from species-level models; large black points and error bars show the mean and 95% confidence intervals across species for each season. Panels display results by window size (columns) and movement metric (rows). Abbreviations: pairwise displacement (PD); net displacement (ND); enhanced vegetation index (EVI).

**
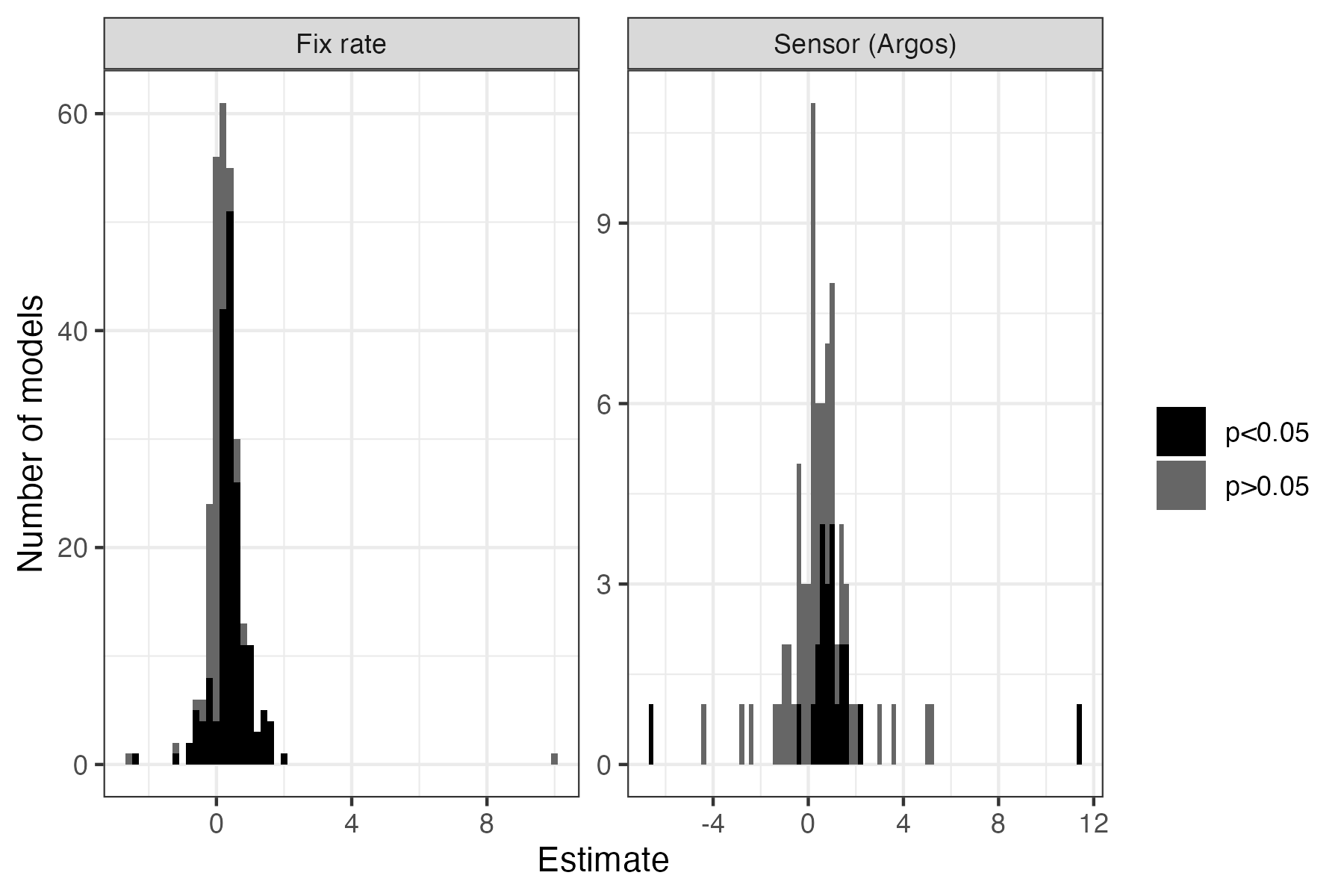
**

***Figure S7:*** Coefficient estimates for fix rate (left) and sensor type (right). A positive value indicates that observed movement distances of a given waterfowl species in a given season were greater for bird-windows with more fixes or those using Argos sensors. Shading indicates statistical significance at p<0.05. All models included a term for fix rate, but only data sets with both GPS and Argos sensors included a term for sensor type. Coefficients are shown for all window sizes, seasons, and movement metrics.


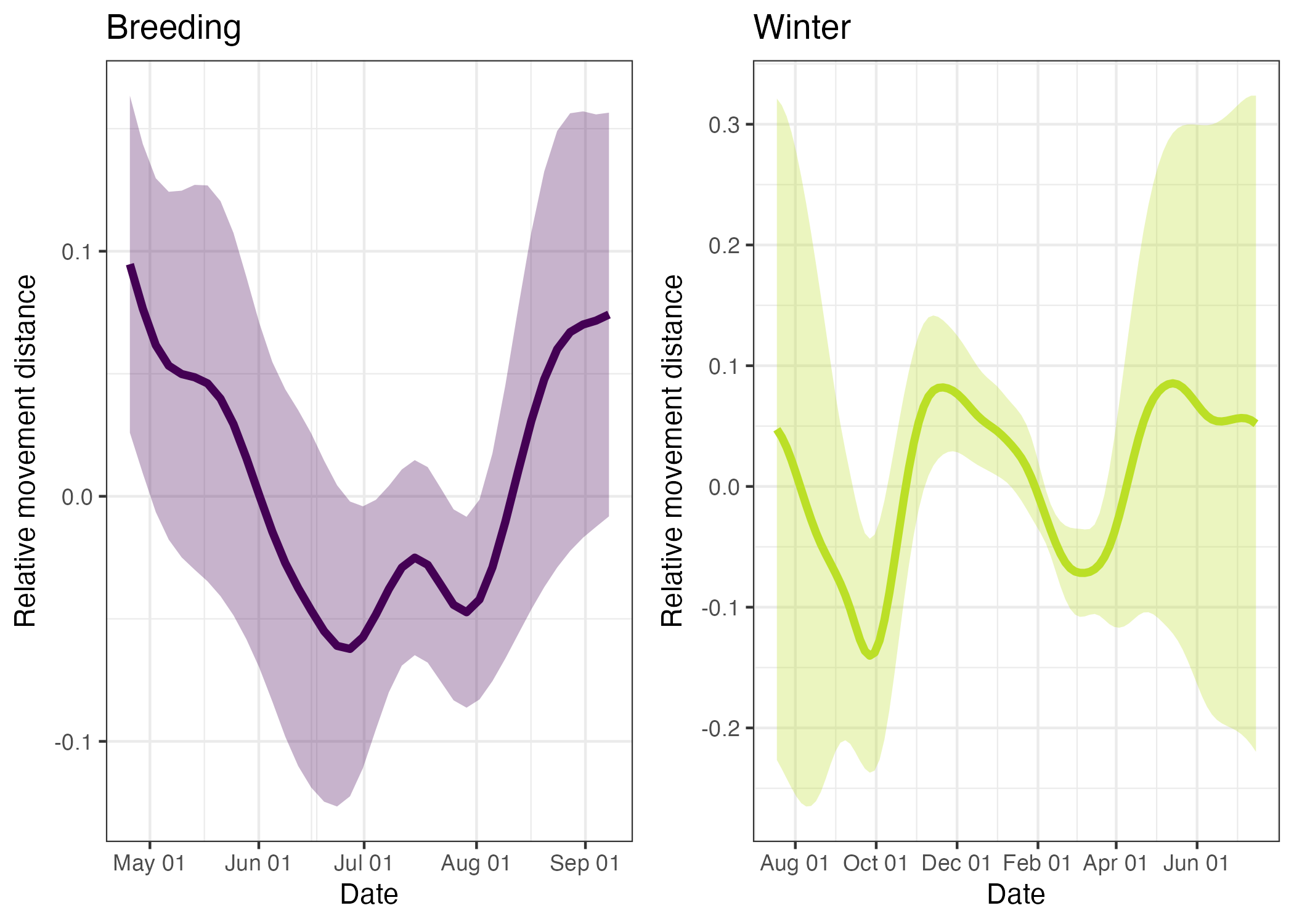


***Figure S8****:* Average effects of day of year in the breeding season (left) and winter (right) on relative waterfowl movement distances. The y-axis shows relative movement distance, e.g., -0.1 represents a reduction in movement of 10% relative to a species’ mean observed movement distance. Note the different scales of the y-axes across panels. Shaded areas show 95% confidence intervals of the mean across species. Only models for 12-hour maximum pairwise displacement are included in the average.


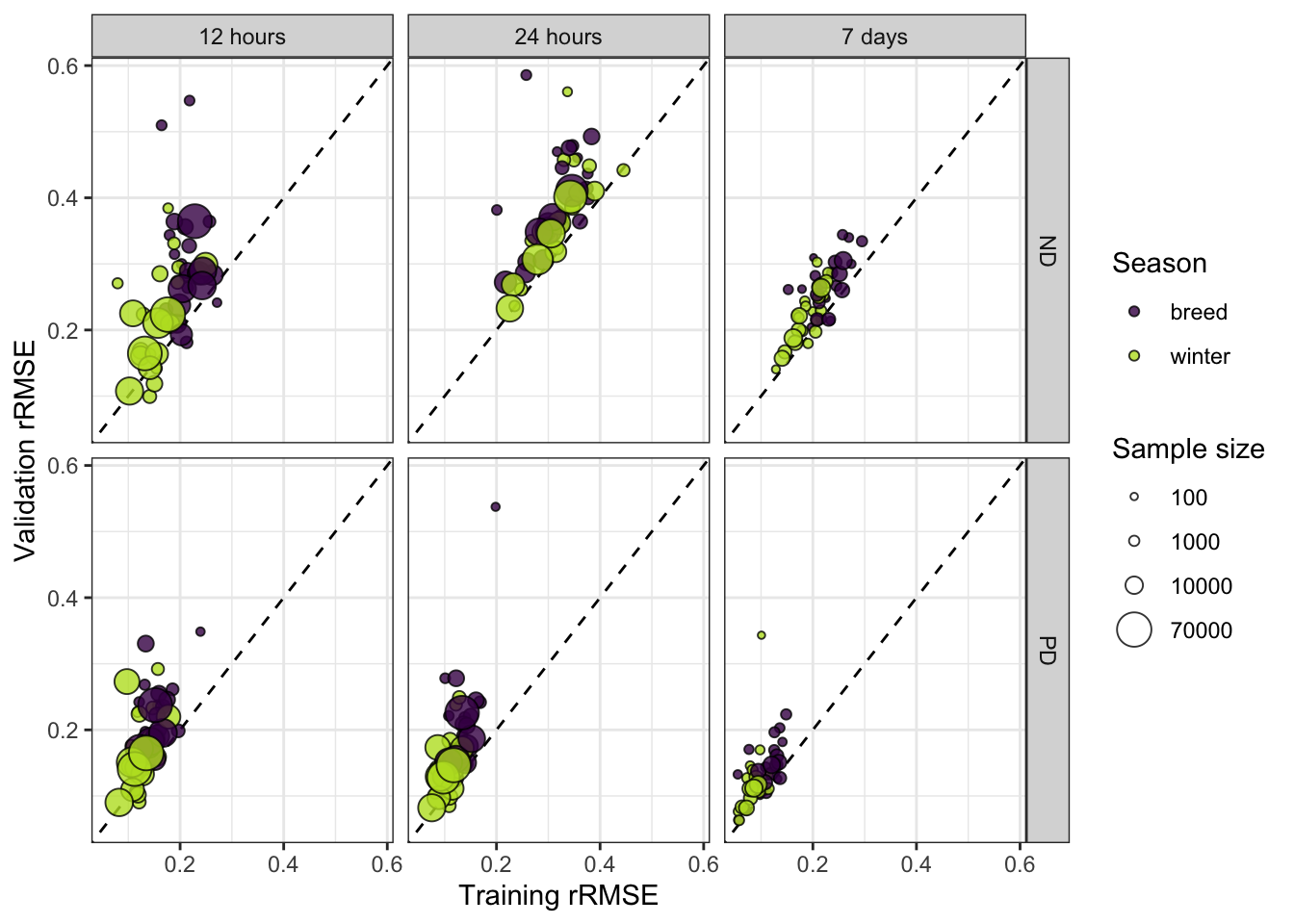


**Figure S9:** Model performance metrics for training and validation data for models of waterfowl movement distances. Relative root mean squared error (rRMSE) is the root mean sqared error standardized by the mean of the data, so that metrics are comparable across models. rRMSE was calculated for training and validation data separately. Lower values of rRMSE indicate better model performance. The dashed line shows a 1:1 relationship between training and validation rRMSE. Columns show window sizes; rows show response variables. Abbreviations: pairwise displacement (PD); net displacement (ND).

***
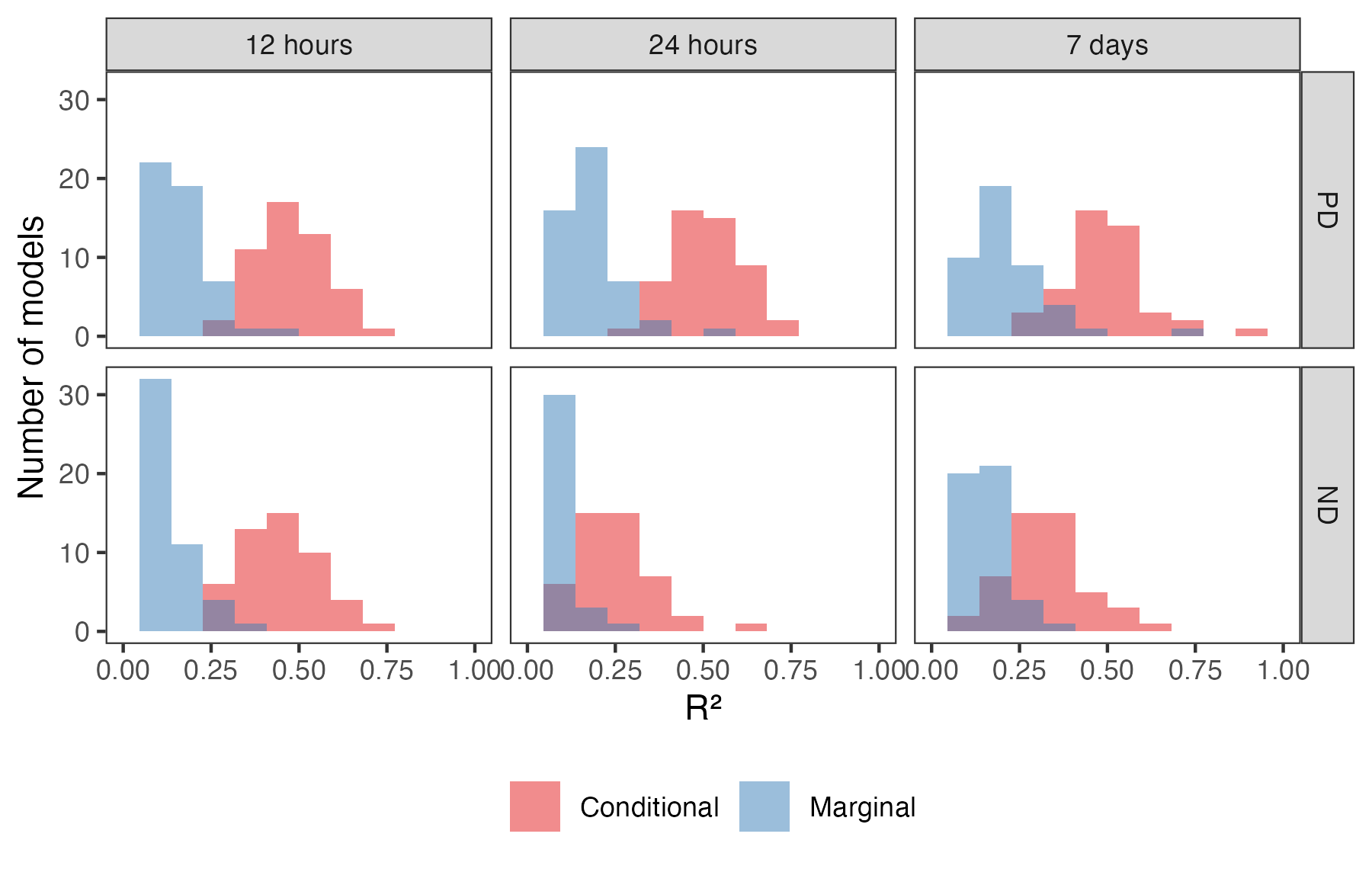
***

***Figure S10***: Coefficients of determination (R^2^) for all models. Conditional R^2^ values (red) are the variance explained by all terms; marginal R^2^ values (blue) include fixed effects only (i.e., exclude terms for space, individual ID, and date). Columns show window sizes; rows show response variables. Abbreviations: pairwise displacement (PD); net displacement (ND).

***
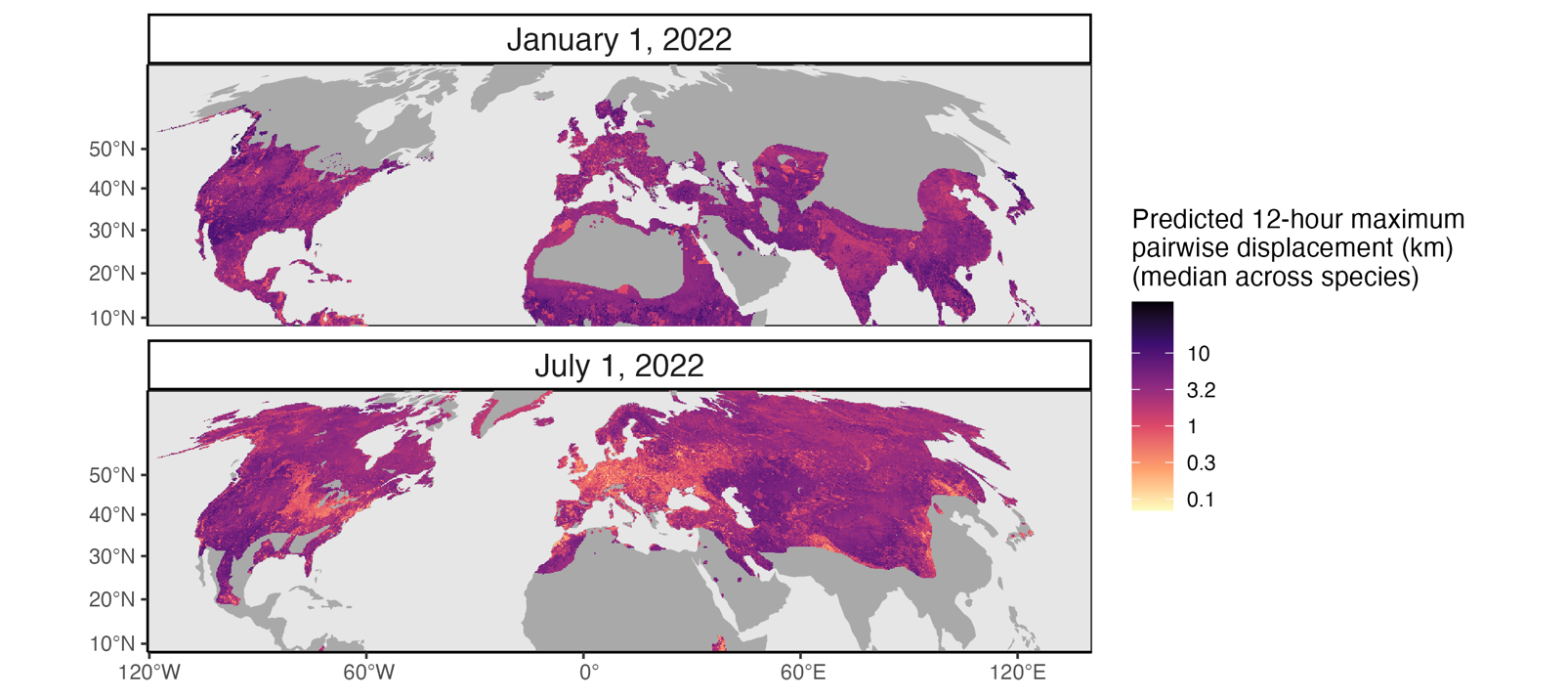
***

***Figure S11***: Median predicted maximum displacement based on environmental conditions on January 1, and July 1, 2022. Figures S8 and Figures S9 display environmental covariates. Predictions are marginal (i.e., do not consider random effects) and distribution-independent (i.e., predictions are made for each species across the entire hemisphere, regardless of their true range). Maps are limited to seasonal ranges of all modeled species for each season, derived from seasonal species range maps from BirdLife International (BirdLife International & Handbook of the Birds of the World 2023). Map in Mollweide projection. Basemap of continental boundaries from Natural Earth.


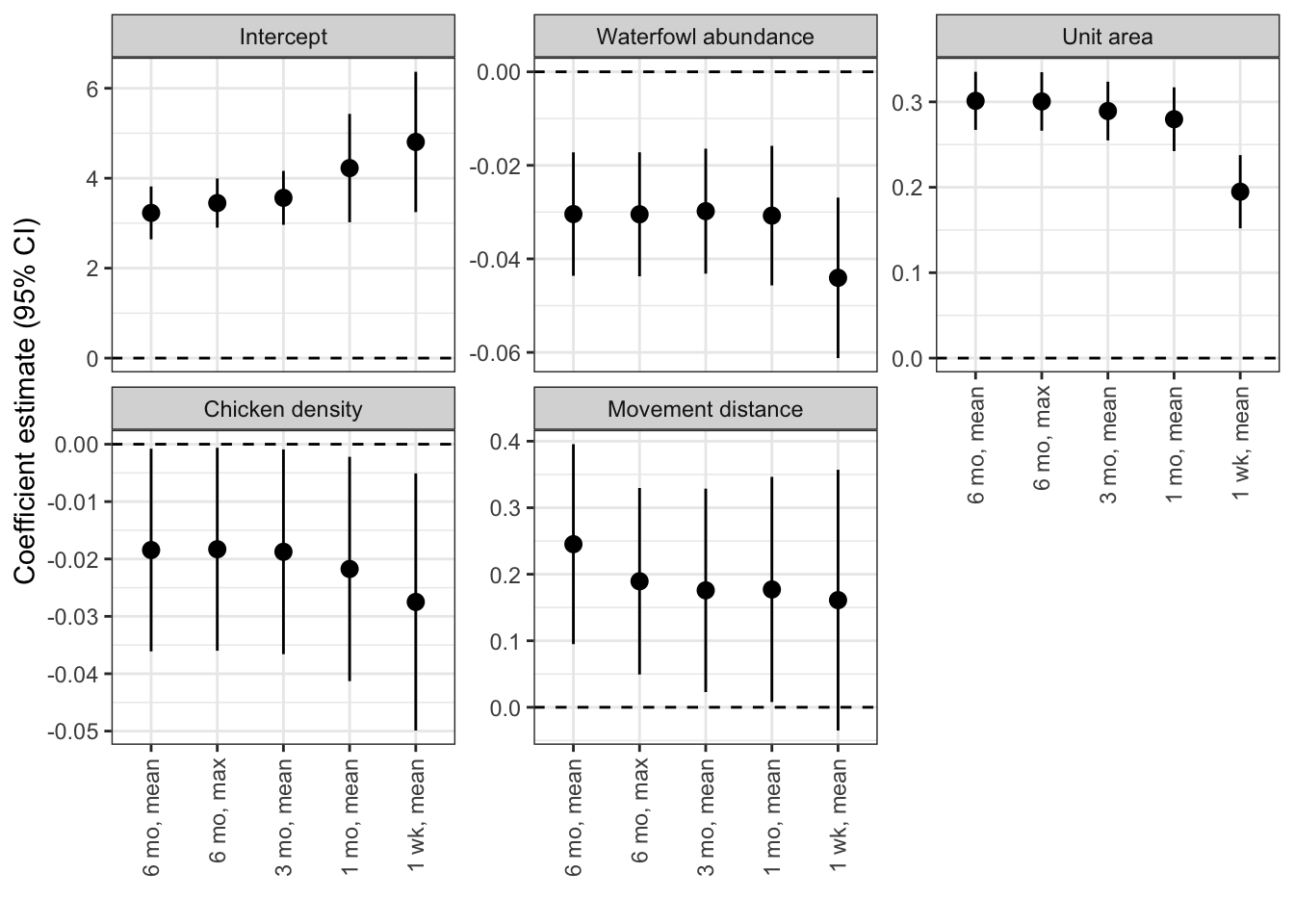


**Figure S12:** Sensitivity of HPAIV spread distance to model assumptions. The y-axis shows the mean and 95% confidence interval of the estimated coefficient from a generalized additive model of spread distance. Each panel shows a different covariate (note the different scales across panels). Each point and range shows results from a model in which spread distance and/or movement distance was calculated differently; spread distance was based on assumptions of 6-month, 3-month, 1-month, or 1-week viral persistence. Movement distance was based on mean or maximum estimated movements across species. The first bar in each plot (6 mo, mean) displays results presented in the main text.

**Table S1**: Permits, funding information, data access instructions, and acknowledgements for data sets used in this study. Where raw data are not published for due to conservation restrictions, data are available upon reasonable request to the listed contact; potential data users are strongly encouraged to collaborate with data contacts, regardless of data publication status. Where data are already published, code is provided to subset data to those used in this analysis. This table is provided as a separate spreadsheet.

**Table S2:** GPS telemetry data used in this study, summarized by species. The number of bird-days is the number of windows included in 24-hour movement analyses. Some species may have more birds included in 12-hour or 7-day analyses. Species are arranged taxonomically (dabbling ducks, diving ducks, geese, shelduck, swans). Continents listed are those for which tracking data were used in this study; some species are present, but not tracked, on other continents.

| **Common name** | **Scientific name** | **# ind-ividuals** | **# bird-days** | **Tag types** | **Continents** |
| --- | --- | --- | --- | --- | --- |
| Northern Pintail | *Anas acuta* | 432 | 29624 | GPS, Argos | Asia, Europe, Africa, Americas |
| American Wigeon | *Mareca americana* | 74 | 5032 | GPS | Americas |
| Northern Shoveler | *Spatula clypeata* | 139 | 11554 | GPS, Argos | Asia, Europe, Africa, Americas |
| Eurasian Teal/Green-winged Teal (American) | *Anas crecca* | 267 | 20805 | GPS, Argos | Asia, Europe, Africa, Americas |
| Cinnamon Teal | *Spatula cyanoptera* | 157 | 13959 | GPS | Americas |
| Blue-winged Teal | *Spatula discors* | 52 | 2197 | Argos, GPS | Americas |
| Eurasian Wigeon | *Mareca penelope* | 77 | 5314 | GPS, Argos | Asia, Europe, Americas |
| Mallard | *Anas platyrhynchos* | 1126 | 135595 | GPS, Argos | Europe, Americas, Asia |
| Garganey | *Spatula querquedula* | 49 | 3671 | GPS, Argos | Asia, Africa |
| Gadwall | *Mareca strepera* | 213 | 13743 | GPS, Argos | Asia, Americas, Europe |
| Greater Scaup | *Aythya marila* | 13 | 196 | GPS | Americas |
| Canvasback | *Aythya valisineria* | 60 | 4520 | GPS | Americas |
| Surf Scoter | *Melanitta perspicillata* | 37 | 158 | Argos | Americas |
| Greater White-fronted Goose | *Anser albifrons* | 450 | 96558 | GPS | Asia, Europe, Americas |
| Greylag Goose | *Anser anser* | 137 | 81944 | GPS | Europe, Asia |
| Pink-footed Goose | *Anser brachyrhynchus* | 167 | 48667 | GPS | Europe |
| Swan Goose | *Anser cygnoides* | 64 | 5737 | GPS | Asia |
| Taiga Bean Goose | *Anser fabalis* | 101 | 18200 | GPS, radio | Asia, Europe |
| Bar-headed Goose | *Anser indicus* | 91 | 7617 | GPS, Argos | Asia |
| Barnacle Goose | *Branta leucopsis* | 188 | 59804 | GPS, Argos | Europe |
| Snow Goose | *Anser caerulescens* | 248 | 43445 | GPS | Americas |
| Ross's Goose | *Anser rossii* | 38 | 1951 | GPS | Americas |
| Ruddy Shelduck | *Tadorna ferruginea* | 51 | 6538 | GPS | Asia |
| Trumpeter Swan | *Cygnus buccinator* | 125 | 59172 | GPS | Americas |
| Tundra Swan | *Cygnus columbianus* | 141 | 27579 | GPS, Argos | Europe, Americas |
| Whooper Swan | *Cygnus cygnus* | 109 | 1820 | GPS, Argos | Asia, Europe, Americas |

**Table S3:** Median observed (raw) movement distances by species. Distances (in km) are reported by window size, season, and metric. ND = net displacement; PD = pairwise displacement. Missing values indicate that a species had insufficient data to be included in models for a given season-window-metric combination.

| **Species** | **12 hours** | | | | **24 hours** | | | | **7 days** | | | |
| --- | --- | --- | --- | --- | --- | --- | --- | --- | --- | --- | --- | --- |
|  | **Breeding** | | **Winter** | | **Breeding** | | **Winter** | | **Breeding** | | **Winter** | |
|  | **ND** | **PD** | **ND** | **PD** | **ND** | **PD** | **ND** | **PD** | **ND** | **PD** | **ND** | **PD** |
| Northern Pintail | 1.39 | 2.21 | 3.82 | 4.63 | 0.61 | 2.93 | 0.85 | 6.48 | 4.18 | 16 | 8.79 | 21.24 |
| American Wigeon | 1.03 | 1.73 | 3.57 | 3.95 | 0.55 | 2.17 | 0.72 | 5.42 | 1.81 | 6.84 | 5.13 | 13.81 |
| Northern Shoveler | 0.56 | 1.02 | 1.49 | 1.93 | 0.21 | 1.4 | 0.3 | 2.44 | 0.97 | 4.32 | 2.34 | 7.45 |
| Eurasian Teal | 0.38 | 0.7 | 1.45 | 1.74 | 0.09 | 0.9 | 0.09 | 2.13 | 0.45 | 2.39 | 1.47 | 4.15 |
| Cinnamon Teal | 0.56 | 0.87 | 0.88 | 1.15 | 0.16 | 1.15 | 0.14 | 1.47 | 0.8 | 3.02 | 1.03 | 3.62 |
| Blue-winged Teal | 0.66 | 1.12 | 1.31 | 1.89 |  | 1.23 |  | 2.15 |  |  |  |  |
| Eurasian Wigeon | 0.39 | 0.76 | 0.63 | 0.86 | 0.17 | 1.21 | 0.19 | 1.19 | 0.63 | 3.55 | 0.96 | 3.86 |
| Mallard | 0.51 | 1 | 1.37 | 1.75 | 0.15 | 1.45 | 0.2 | 2.5 | 0.78 | 3.63 | 1.93 | 5.8 |
| Garganey | 0.61 | 0.93 | 2.07 | 2.6 | 0.15 | 1.22 | 0.14 | 3.37 | 1.23 | 7.04 | 2.06 | 7.1 |
| Gadwall | 0.49 | 0.8 | 1.48 | 1.97 | 0.26 | 1.16 | 0.24 | 2.56 | 0.9 | 3.37 | 1.63 | 5.76 |
| Greater Scaup | 0.61 | 0.98 |  |  |  | 1.15 |  |  | 1.64 |  |  |  |
| Canvasback | 0.63 | 1 | 1.82 | 2.55 | 0.51 | 1.9 | 0.83 | 3.7 | 1.07 |  | 3.4 | 10.34 |
| Surf Scoter |  | 4.31 |  |  |  | 4.95 |  |  | 5.28 |  |  |  |
| Greater White-fronted Goose | 0.47 | 0.79 | 2.76 | 3.56 | 0.43 | 1.23 | 1.22 | 4.92 | 1.24 | 3.91 | 4.64 | 12.58 |
| Greylag Goose | 0.84 | 1.81 | 1.59 | 2.42 | 0.43 | 2.44 | 0.41 | 3.07 | 1.48 | 5.02 | 2.31 | 6.92 |
| Pink-footed Goose | 0.74 | 1.03 | 4.43 | 4.89 | 0.72 | 1.51 | 0.79 | 6.39 | 1.79 | 4.25 | 8.05 | 17.75 |
| Swan Goose | 1.31 | 2.27 | 1.53 | 2.15 | 0.7 | 3.03 | 1.07 | 3.41 | 3.02 | 10.26 | 4.55 | 13.12 |
| Taiga Bean Goose | 0.8 | 1.29 | 3.28 | 3.77 | 0.67 | 1.99 | 0.44 | 4.51 | 1.87 | 5.19 | 3.44 | 7.79 |
| Bar-headed Goose | 1.3 | 2.08 | 3.24 | 5.2 | 1.26 | 3.99 | 0.81 | 7.46 | 3.41 | 16.97 | 4.78 | 17.9 |
| Barnacle Goose | 0.53 | 0.88 | 2.63 | 3.36 | 0.44 | 1.35 | 1.04 | 4.49 | 1.79 | 4.51 | 4.59 | 11.71 |
| Snow Goose | 0.85 | 1.25 | 6.13 | 9.22 | 1.23 | 2.29 | 3.71 | 13.77 | 4.26 | 8.58 | 16.65 | 46.54 |
| Ross's Goose | 0.77 | 1.25 | 3.81 | 6.1 | 1.09 | 2.26 | 2.93 | 9.45 | 3.46 | 6.68 | 11.12 | 29.98 |
| Ruddy Shelduck | 1.89 | 4.01 | 2.19 | 4.57 | 1.11 | 6.66 | 1.19 | 6.82 | 4.1 | 19.91 | 4.04 | 19.53 |
| Trumpeter Swan | 0.37 | 0.93 | 1.08 | 1.75 | 0.2 | 1.32 | 0.32 | 3.9 | 0.53 | 2.82 | 2.9 | 11.04 |
| Tundra Swan | 0.4 | 0.86 | 3.4 | 3.71 | 0.41 | 1.25 | 0.4 | 5.42 | 1.46 | 5.49 | 6.52 | 14.46 |
| Whooper Swan | 1.07 | 1.55 | 1.61 | 1.98 | 0.81 | 1.82 | 0.54 | 2.5 | 1.84 | 4.48 | 0.94 | 2.98 |

**Table S4**: Model results for gross effect size. Reference levels are: 12 hours, breeding season, net displacement, dabbling duck. Each set of results is from a generalized linear mixed-effects model of gross effect size as a function of species group (goose, swan, dabbling duck, diving duck, shelduck), window size, season, and response variable. Models also included a random slope for each species-season combination. Abbreviations: pairwise displacement (PD); enhanced vegetation index (EVI); standard deviation (SD).

| **Environmental variable** | **Term** | **Estimate** | **Std. Error** | ***z*** | ***p*** |
| --- | --- | --- | --- | --- | --- |
| Mean EVI | (Intercept) | 0.368 | 0.064 | 5.781 | <0.001 |
|  | winter | -0.041 | 0.061 | -0.667 | 0.505 |
|  | 24 hours | -0.043 | 0.040 | -1.089 | 0.276 |
|  | 7 days | -0.028 | 0.040 | -0.704 | 0.481 |
|  | PD | -0.022 | 0.033 | -0.676 | 0.499 |
|  | diving duck | 0.089 | 0.123 | 0.722 | 0.470 |
|  | shelduck | -0.074 | 0.159 | -0.465 | 0.642 |
|  | goose | -0.026 | 0.070 | -0.371 | 0.711 |
|  | swan | 0.166 | 0.100 | 1.662 | 0.096 |
| SD EVI | (Intercept) | 0.410 | 0.059 | 6.969 | <0.001 |
|  | winter | -0.062 | 0.058 | -1.070 | 0.285 |
|  | 24 hours | 0.006 | 0.037 | 0.162 | 0.872 |
|  | 7 days | 0.002 | 0.039 | 0.062 | 0.950 |
|  | PD | -0.041 | 0.031 | -1.319 | 0.187 |
|  | diving duck | 0.322 | 0.118 | 2.718 | 0.007 |
|  | shelduck | 0.026 | 0.144 | 0.177 | 0.859 |
|  | goose | 0.011 | 0.067 | 0.157 | 0.875 |
|  | swan | -0.188 | 0.092 | -2.036 | 0.042 |
| Croplands | (Intercept) | 0.962 | 0.092 | 10.500 | <0.001 |
|  | winter | -0.156 | 0.088 | -1.776 | 0.076 |
|  | 24 hours | -0.110 | 0.077 | -1.431 | 0.152 |
|  | 7 days | -0.294 | 0.078 | -3.782 | <0.001 |
|  | PD | -0.064 | 0.063 | -1.013 | 0.311 |
|  | diving duck | 0.312 | 0.199 | 1.572 | 0.116 |
|  | shelduck | 0.386 | 0.194 | 1.992 | 0.046 |
|  | goose | 0.392 | 0.102 | 3.835 | <0.001 |
|  | swan | 0.098 | 0.131 | 0.746 | 0.455 |
| Land cover diversity | (Intercept) | 0.718 | 0.098 | 7.339 | <0.001 |
|  | winter | 0.260 | 0.096 | 2.724 | 0.006 |
|  | 24 hours | -0.038 | 0.059 | -0.650 | 0.516 |
|  | 7 days | -0.206 | 0.060 | -3.428 | 0.001 |
|  | PD | 0.019 | 0.049 | 0.398 | 0.691 |
|  | diving duck | 0.168 | 0.191 | 0.883 | 0.377 |
|  | shelduck | 0.294 | 0.246 | 1.197 | 0.231 |
|  | goose | 0.094 | 0.109 | 0.862 | 0.389 |
|  | swan | 0.248 | 0.155 | 1.605 | 0.109 |
| Protected areas | (Intercept) | 0.492 | 0.065 | 7.578 | <0.001 |
|  | winter | 0.213 | 0.062 | 3.422 | 0.001 |
|  | 24 hours | -0.020 | 0.040 | -0.496 | 0.620 |
|  | 7 days | -0.195 | 0.041 | -4.797 | <0.001 |
|  | PD | -0.011 | 0.033 | -0.335 | 0.737 |
|  | diving duck | 0.269 | 0.127 | 2.129 | 0.033 |
|  | shelduck | -0.056 | 0.160 | -0.350 | 0.726 |
|  | goose | 0.101 | 0.071 | 1.419 | 0.156 |
|  | swan | 0.352 | 0.101 | 3.484 | <0.001 |
| Surface water | (Intercept) | 0.543 | 0.082 | 6.609 | <0.001 |
|  | winter | 0.074 | 0.077 | 0.959 | 0.338 |
|  | 24 hours | -0.011 | 0.056 | -0.193 | 0.847 |
|  | 7 days | -0.138 | 0.057 | -2.423 | 0.015 |
|  | PD | -0.024 | 0.046 | -0.517 | 0.605 |
|  | diving duck | 0.265 | 0.157 | 1.690 | 0.091 |
|  | shelduck | -0.047 | 0.200 | -0.236 | 0.813 |
|  | goose | 0.175 | 0.088 | 1.983 | 0.047 |
|  | swan | -0.159 | 0.126 | -1.263 | 0.207 |
| Human pop. density (log10) | (Intercept) | 1.229 | 0.123 | 9.970 | <0.001 |
|  | winter | -0.438 | 0.126 | -3.486 | <0.001 |
|  | 24 hours | -0.107 | 0.059 | -1.809 | 0.070 |
|  | 7 days | -0.391 | 0.060 | -6.532 | <0.001 |
|  | PD | -0.144 | 0.049 | -2.972 | 0.003 |
|  | diving duck | -0.562 | 0.277 | -2.032 | 0.042 |
|  | shelduck | -0.147 | 0.323 | -0.455 | 0.649 |
|  | goose | 0.087 | 0.142 | 0.612 | 0.540 |
|  | swan | 0.181 | 0.203 | 0.891 | 0.373 |
| Precipitation (log10) | (Intercept) | 0.059 | 0.017 | 3.562 | <0.001 |
|  | winter | 0.017 | 0.015 | 1.149 | 0.250 |
|  | 24 hours | 0.029 | 0.013 | 2.161 | 0.031 |
|  | 7 days | 0.051 | 0.013 | 3.800 | <0.001 |
|  | PD | -0.045 | 0.011 | -4.130 | <0.001 |
|  | diving duck | 0.084 | 0.030 | 2.760 | 0.006 |
|  | shelduck | 0.054 | 0.038 | 1.423 | 0.155 |
|  | goose | -0.013 | 0.017 | -0.764 | 0.445 |
|  | swan | -0.022 | 0.024 | -0.918 | 0.358 |
| Temperature | (Intercept) | 0.255 | 0.046 | 5.532 | <0.001 |
|  | winter | -0.052 | 0.042 | -1.236 | 0.216 |
|  | 24 hours | -0.005 | 0.034 | -0.151 | 0.880 |
|  | 7 days | 0.044 | 0.034 | 1.297 | 0.195 |
|  | PD | -0.049 | 0.028 | -1.761 | 0.078 |
|  | diving duck | -0.148 | 0.087 | -1.705 | 0.088 |
|  | shelduck | -0.034 | 0.109 | -0.306 | 0.760 |
|  | goose | 0.005 | 0.048 | 0.110 | 0.913 |
|  | swan | 0.144 | 0.069 | 2.088 | 0.037 |
| Wind speed | (Intercept) | 0.076 | 0.028 | 2.671 | 0.008 |
|  | winter | 0.033 | 0.026 | 1.276 | 0.202 |
|  | 24 hours | 0.021 | 0.021 | 0.995 | 0.320 |
|  | 7 days | 0.044 | 0.021 | 2.082 | 0.037 |
|  | PD | -0.032 | 0.017 | -1.877 | 0.060 |
|  | diving duck | 0.054 | 0.054 | 1.000 | 0.317 |
|  | shelduck | 0.086 | 0.068 | 1.267 | 0.205 |
|  | goose | 0.028 | 0.030 | 0.953 | 0.341 |
|  | swan | 0.013 | 0.043 | 0.305 | 0.760 |

**Table S5:** Model results for net effect size. Reference levels are: 12 hours, breeding season, net displacement, dabbling duck. Each set of results is from a generalized linear mixed-effects model of net effect size as a function of species group (goose, swan, dabbling duck, diving duck), window size, season, and response variable. Models also included a random slope for each species-season combination. Abbreviations: pairwise displacement (PD); enhanced vegetation index (EVI); standard deviation (SD).

| **Environmental variable** | **Term** | **Estimate** | **Std. Error** | ***z*** | ***p*** |
| --- | --- | --- | --- | --- | --- |
| Mean EVI | Intercept | -0.118 | 0.067 | -1.757 | 0.079 |
|  | winter | -0.019 | 0.066 | -0.290 | 0.772 |
|  | 24 hour | 0.033 | 0.038 | 0.878 | 0.380 |
|  | 7 day | 0.116 | 0.038 | 3.021 | 0.003 |
|  | PD | -0.037 | 0.031 | -1.179 | 0.238 |
|  | diving duck | -0.215 | 0.132 | -1.633 | 0.103 |
|  | shelduck | 0.010 | 0.171 | 0.058 | 0.954 |
|  | goose | 0.032 | 0.075 | 0.420 | 0.675 |
|  | swan | -0.213 | 0.108 | -1.978 | 0.048 |
| SD EVI | Intercept | -0.165 | 0.062 | -2.677 | 0.007 |
|  | winter | 0.033 | 0.064 | 0.519 | 0.604 |
|  | 24 hour | 0.028 | 0.032 | 0.867 | 0.386 |
|  | 168 | 0.027 | 0.033 | 0.798 | 0.425 |
|  | PD | 0.001 | 0.027 | 0.037 | 0.970 |
|  | diving duck | -0.040 | 0.126 | -0.317 | 0.751 |
|  | shelduck | -0.107 | 0.158 | -0.680 | 0.497 |
|  | goose | -0.077 | 0.073 | -1.055 | 0.291 |
|  | swan | 0.070 | 0.100 | 0.698 | 0.485 |
| Croplands | Intercept | -0.198 | 0.147 | -1.344 | 0.179 |
|  | winter | -0.184 | 0.164 | -1.122 | 0.262 |
|  | 24 hour | 0.072 | 0.068 | 1.052 | 0.293 |
|  | 168 | 0.249 | 0.069 | 3.595 | <0.001 |
|  | PD | -0.021 | 0.056 | -0.368 | 0.713 |
|  | diving duck | -0.501 | 0.374 | -1.342 | 0.180 |
|  | shelduck | -0.546 | 0.372 | -1.468 | 0.142 |
|  | goose | -0.249 | 0.190 | -1.313 | 0.189 |
|  | swan | 0.261 | 0.251 | 1.038 | 0.299 |
| Land cover diversity | Intercept | -0.571 | 0.106 | -5.364 | <0.001 |
|  | winter | -0.376 | 0.106 | -3.553 | <0.001 |
|  | 24 hour | 0.052 | 0.058 | 0.891 | 0.373 |
|  | 7 day | 0.255 | 0.059 | 4.300 | <0.001 |
|  | PD | -0.002 | 0.048 | -0.033 | 0.974 |
|  | diving duck | -0.134 | 0.210 | -0.637 | 0.524 |
|  | shelduck | -0.237 | 0.273 | -0.870 | 0.384 |
|  | goose | -0.084 | 0.120 | -0.699 | 0.484 |
|  | swan | -0.237 | 0.172 | -1.381 | 0.167 |
| Protected areas | Intercept | -0.138 | 0.080 | -1.721 | 0.085 |
|  | winter | -0.325 | 0.082 | -3.987 | <0.001 |
|  | 24 hour | 0.011 | 0.036 | 0.321 | 0.748 |
|  | 7 day | 0.067 | 0.036 | 1.839 | 0.066 |
|  | PD | -0.025 | 0.030 | -0.862 | 0.388 |
|  | diving duck | -0.008 | 0.162 | -0.049 | 0.961 |
|  | shelduck | 0.045 | 0.211 | 0.212 | 0.832 |
|  | goose | -0.007 | 0.093 | -0.080 | 0.936 |
|  | swan | -0.204 | 0.133 | -1.538 | 0.124 |
| Surface water | Intercept | -0.047 | 0.111 | -0.420 | 0.675 |
|  | winter | 0.062 | 0.112 | 0.552 | 0.581 |
|  | 24 hour | 0.009 | 0.055 | 0.169 | 0.866 |
|  | 7 day | 0.110 | 0.056 | 1.970 | 0.049 |
|  | PD | -0.076 | 0.045 | -1.682 | 0.093 |
|  | diving duck | 0.020 | 0.222 | 0.090 | 0.929 |
|  | shelduck | -0.432 | 0.290 | -1.490 | 0.136 |
|  | goose | -0.371 | 0.128 | -2.907 | 0.004 |
|  | swan | -0.107 | 0.182 | -0.584 | 0.559 |
| Human pop. density (log10) | Intercept | -1.030 | 0.150 | -6.852 | <0.001 |
|  | winter | 0.551 | 0.156 | 3.527 | <0.001 |
|  | 24 hour | 0.134 | 0.059 | 2.288 | 0.022 |
|  | 7 day | 0.421 | 0.060 | 7.038 | <0.001 |
|  | PD | 0.093 | 0.048 | 1.918 | 0.055 |
|  | diving duck | 0.486 | 0.342 | 1.420 | 0.156 |
|  | shelduck | 0.149 | 0.403 | 0.371 | 0.711 |
|  | goose | 0.010 | 0.177 | 0.054 | 0.957 |
|  | swan | -0.154 | 0.253 | -0.610 | 0.542 |
| Precipitation (log10) | Intercept | 0.005 | 0.018 | 0.302 | 0.763 |
|  | winter | 0.031 | 0.016 | 1.987 | 0.047 |
|  | 24 hour | 0.023 | 0.014 | 1.603 | 0.109 |
|  | 7 day | 0.039 | 0.014 | 2.730 | 0.006 |
|  | PD | -0.025 | 0.012 | -2.171 | 0.030 |
|  | diving duck | -0.019 | 0.033 | -0.572 | 0.567 |
|  | shelduck | 0.048 | 0.041 | 1.170 | 0.242 |
|  | goose | -0.037 | 0.018 | -2.075 | 0.038 |
|  | swan | -0.018 | 0.026 | -0.721 | 0.471 |
| Temperature | Intercept | -0.044 | 0.046 | -0.956 | 0.339 |
|  | winter | 0.055 | 0.042 | 1.296 | 0.195 |
|  | 24 hour | -0.071 | 0.034 | -2.098 | 0.036 |
|  | 7 day | -0.053 | 0.034 | -1.550 | 0.121 |
|  | PD | 0.034 | 0.028 | 1.232 | 0.218 |
|  | diving duck | 0.045 | 0.086 | 0.527 | 0.598 |
|  | shelduck | 0.113 | 0.109 | 1.037 | 0.300 |
|  | goose | -0.004 | 0.048 | -0.091 | 0.927 |
|  | swan | 0.258 | 0.068 | 3.765 | <0.001 |
| Wind speed | Intercept | 0.025 | 0.030 | 0.855 | 0.393 |
|  | winter | -0.019 | 0.027 | -0.706 | 0.480 |
|  | 24 hour | 0.001 | 0.021 | 0.042 | 0.967 |
|  | 7 day | 0.019 | 0.022 | 0.863 | 0.388 |
|  | PD | -0.008 | 0.018 | -0.464 | 0.643 |
|  | diving duck | -0.101 | 0.056 | -1.817 | 0.069 |
|  | shelduck | -0.193 | 0.071 | -2.730 | 0.006 |
|  | goose | -0.113 | 0.031 | -3.624 | <0.001 |
|  | swan | -0.055 | 0.044 | -1.244 | 0.214 |

**Table S6:** Results from a generalized additive model of HPAIV spread distance (log_10_-transformed). The model also contained a smoothed term for date by continent (Europe, Asia, or North America). Reference level for continent is Americas. Movement distance, waterfowl abundance, ADM-2 area, and chicken density were log_10_-transformed.

| **Term** | **Estimate** | **Std. Error** | ***t*** | ***p*** |
| --- | --- | --- | --- | --- |
| Intercept | 3.229 | 0.300 | 10.774 | <0.001 |
| Region (Asia) | 1.143 | 0.461 | 2.481 | 0.013 |
| Region (Europe) | -0.224 | 0.048 | -4.659 | <0.001 |
| Mean movement distance | 0.245 | 0.077 | 3.198 | 0.001 |
| Waterfowl abundance | -0.030 | 0.007 | -4.516 | <0.001 |
| ADM-2 area | 0.301 | 0.017 | 17.359 | <0.001 |
| Chicken density | -0.018 | 0.009 | -2.047 | 0.041 |
